# Correction: Selection of Motor Programs for Suppressing Food Intake and Inducing Locomotion in the Drosophila Brain

**DOI:** 10.1371/journal.pbio.1002016

**Published:** 2014-11-06

**Authors:** The PLOS Biology Staff

## Notice of Republication

This article was republished on August 7^th^, 2014 to correct an error in the 7^th^ author’s name, as it appears in the author byline, paragraph 3 of the Discussion, and in Reference #66. The correct name is: Roland Spieß. Please download this article again to view the correct version. The originally published, uncorrected article and the republished, corrected article are provided here for reference.

## Supporting Information

File S1 Originally published, uncorrected article.
(PDF)

File S2 Republished corrected article.
(PDF)

## References

[1] Schoofs A, Hückesfeld S, Schlegel P, Miroschnikow A, Peters M, et al (2014) Selection of Motor Programs for Suppressing Food Intake and Inducing Locomotion in the Drosophila Brain. PLoS Biol 12(6): e1001893 doi:10.1371/journal.pbio.1001893 24960360 10.1371/journal.pbio.1001893 PMC4068981

