# Supplementary figures and images for: Selection of Motor Programs for Suppressing Food Intake and Inducing Locomotion in the Drosophila Brain
Source: PLoS Biol. 2014 Jun 24;12(6):e1001893. doi: 10.1371/journal.pbio.1001893 (PMC4068981; doi:10.1371/journal.pbio.1001893)

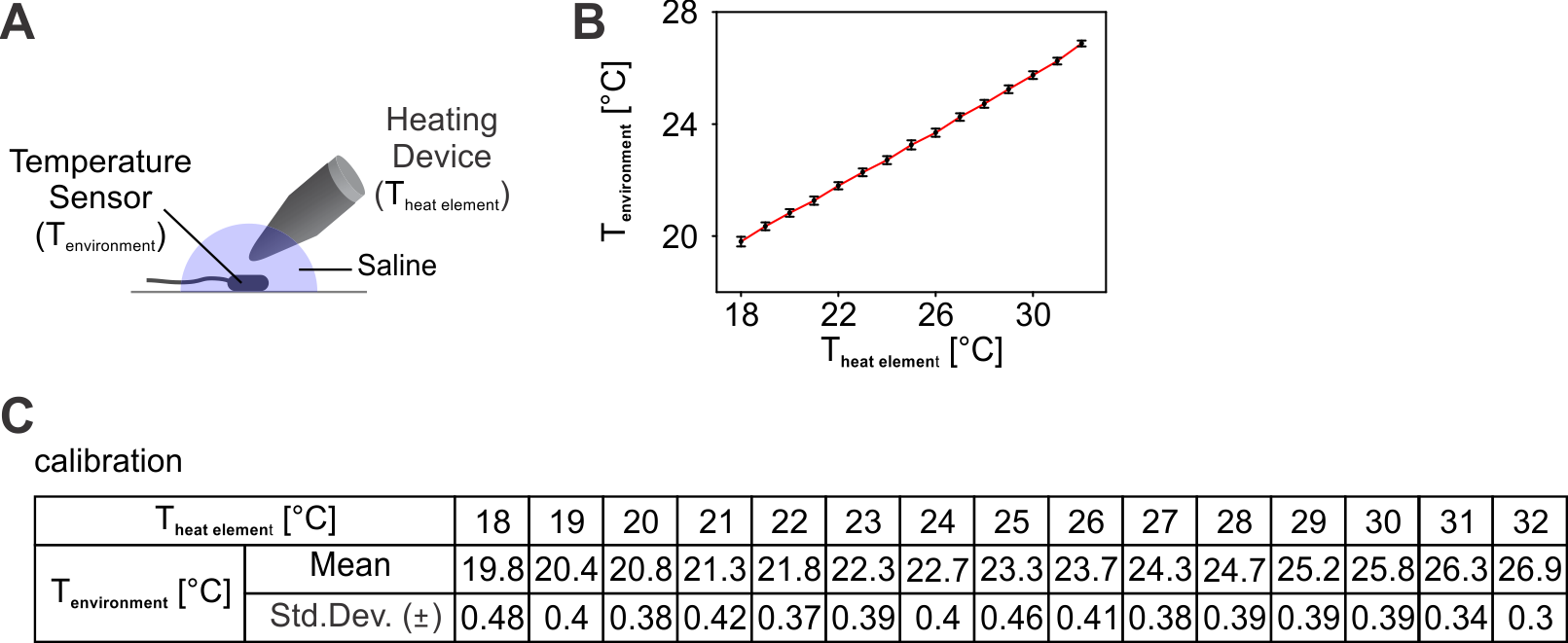

Supplement: Figure S1 — (A) Experimental set up of heating device calibration. (B) Calibration curve of the heating device (x-axis – Theat element [°C], y-axis – Tenvironment [°C]). At Theat element 18°C the measured Tenvironment was 19.8+/−0.48°C and at Theat element 32°C the measured Tenvironment was 26.9+/−0.3°C. (TIF) [file pbio.1001893.s001.tif]

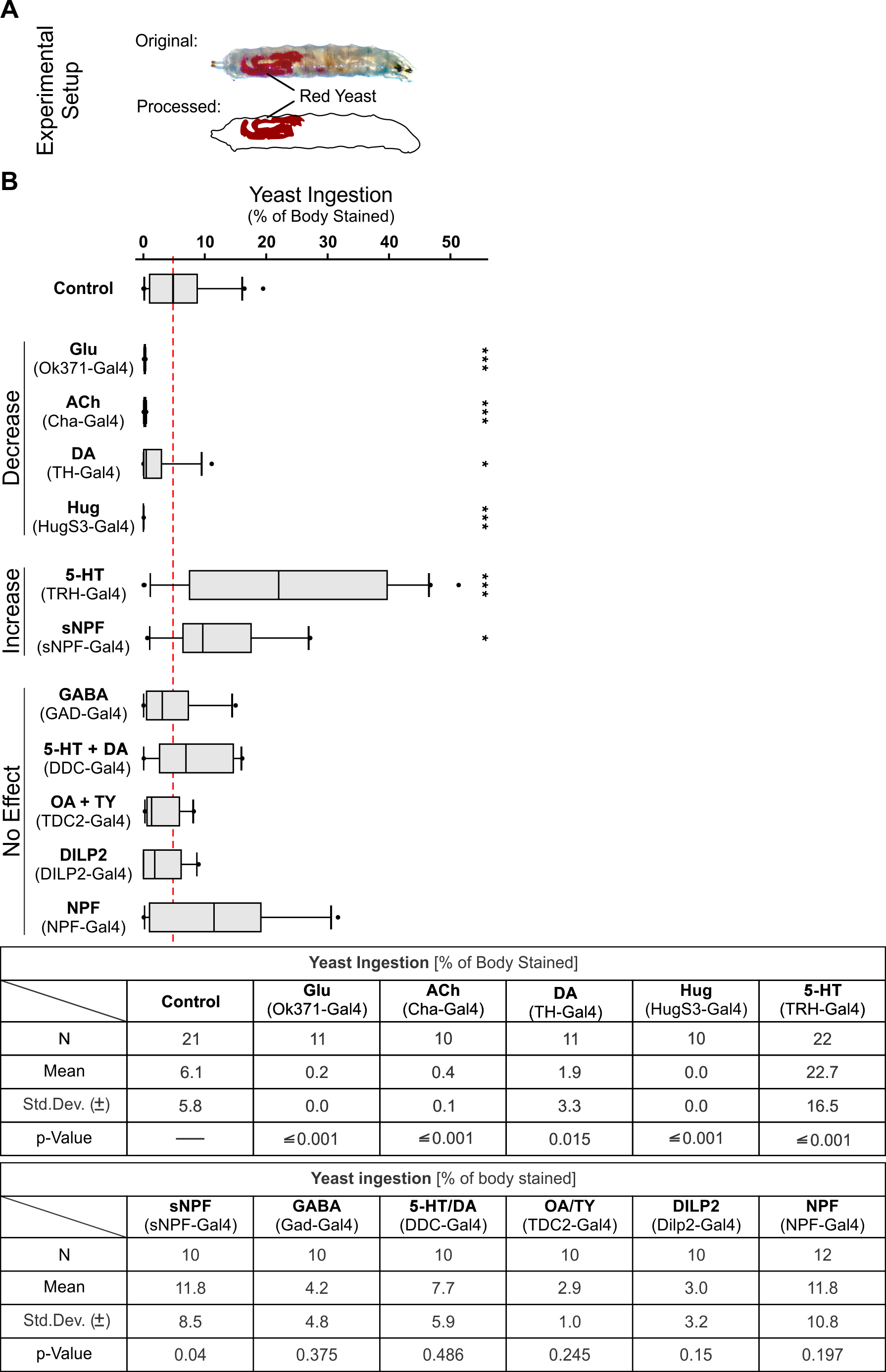

Supplement: Figure S2 — (A) Experimental setup: yeast intake of larvae [% of body stained] was determined after 20 min of dTrpA1-activation. The following major neurotransmitter systems were used for the initial screening: glutamatergic (Glu), cholinergic (ACh), GABAergic (GABA), serotonergic (5-HT), dopaminergic (DA), combined serotonergic/dopaminergic (5-HT/DA) and combined octopaminergic/tyraminergic (OA/TYR) neuronal populations. We also tested four neuropeptide genes shown in earlier studies to be involved in some aspect of feeding response: Drosophila insulin-like peptide (Dilp), hugin (Hug), neuropeptide F (NPF) and short NPF (sNPF) (see Materials and Methods for the respective Gal4-lines). (B) Statistical data of yeast intake screen for all tested Gal4-lines is represented as box plots. Crosses were categorized based on their effect on larval food intake (Mann-Whitney Rank Sum Test: *p≤0.05, **p≤0.01, ***p≤0.001). (TIF) [file pbio.1001893.s002.tif]

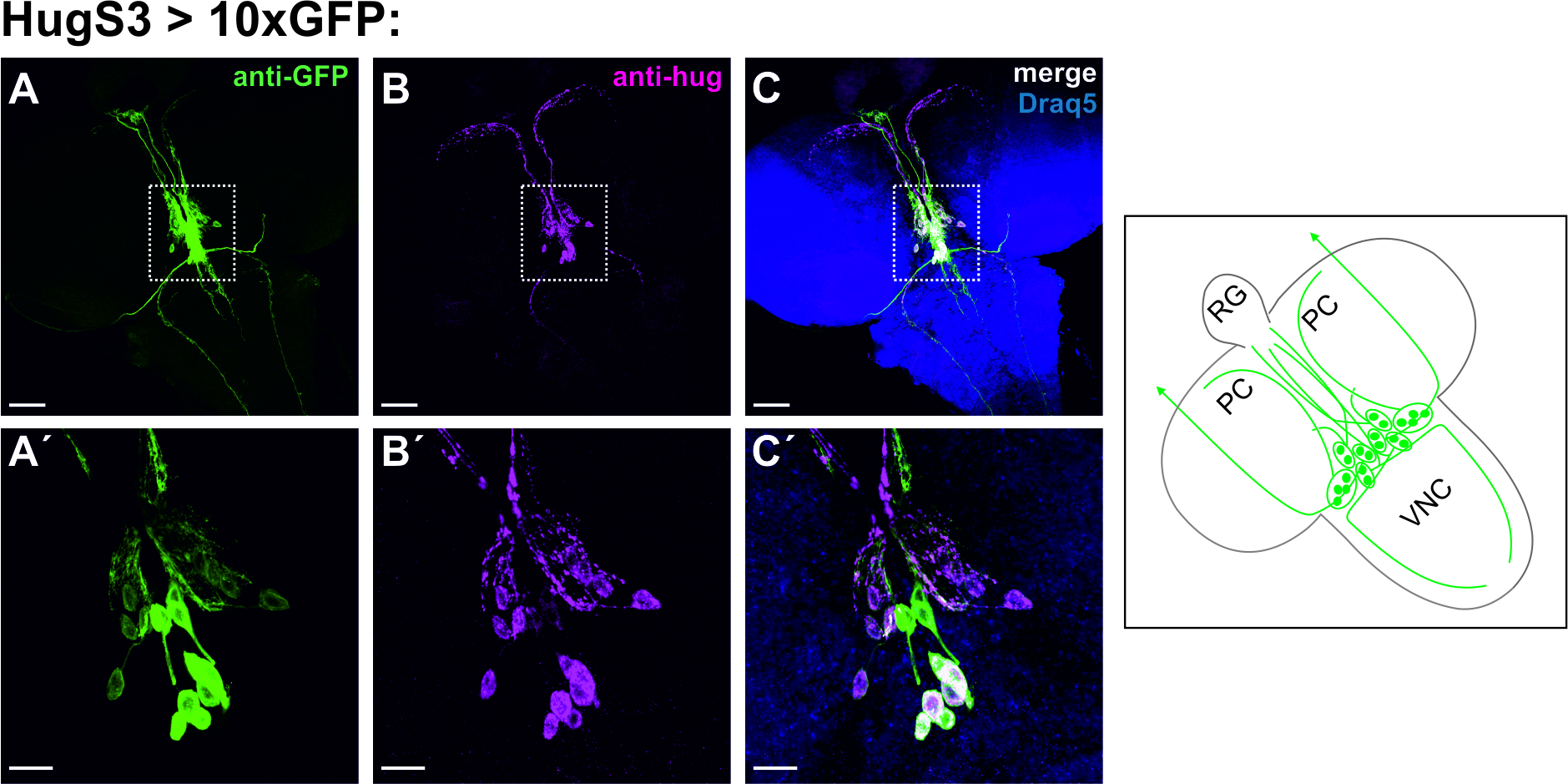

Supplement: Figure S3 — (A–C) Antibody staining of HugS3>10×GFP expression pattern in the CNS; Magnification (A′–C) of hugin cell cluster (20 cells) in the SOG (A: scale bar: 50 µm; A′: scale bar: 10 µm). Schematic summary of the projection pattern HugS3-Gal4 line in the larval CNS (right side). Target region of the projections are: PC, RG, SOG, VNC and periphery via PaN. Abbr.: CNS – central nervous system; PaN – prothoracic accessory nerve; PC – protocerebrum; RG – ring gland; SOG – subesophageal ganglion; VNC – ventral nerve cord. (TIF) [file pbio.1001893.s003.tif]

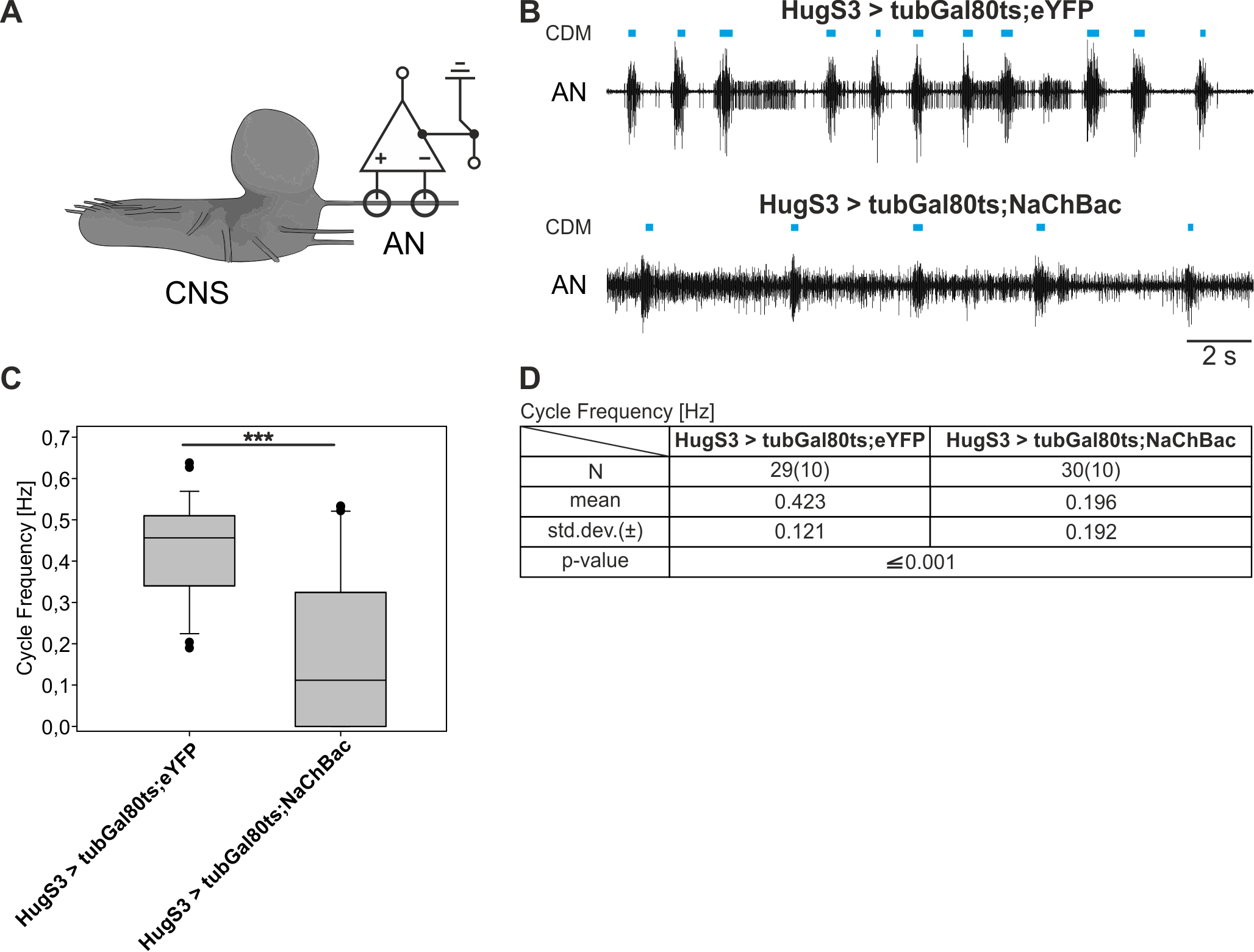

Supplement: Figure S4 — (A) Experimental set up of AN recordings at the isolated CNS. Larvae of both genotypes were 166+/−2 h old (raised on 18°C) and kept for at least 8–12 h on 30°C before recording. (B) Original AN recordings of HugS3>tubGal80ts;eYFP and HugS3>tubGal80ts;NaChBac (colored boxes represent the CDM activity). (C) Box plot of the cycle frequency [Hz] of HugS3>tubGal80ts;eYFP (mean (std. dev.): 0.423 (+/−0.121); number of larvae (number of experiments): 29(10)) and HugS3>tubGal80ts;NaChBac (mean (std. dev.): 0.196 (+/−0.192); number of larvae (number of experiments): 30(10)). HugS3>tubGal80ts;NaChBac was significant different to HugS3>tubGal80ts;eYFP (p-value≤0.001). Abbr.: AN – antennal nerve; CDM – cibarial dilator muscle; CNS – central nervous system. (TIF) [file pbio.1001893.s004.tif]

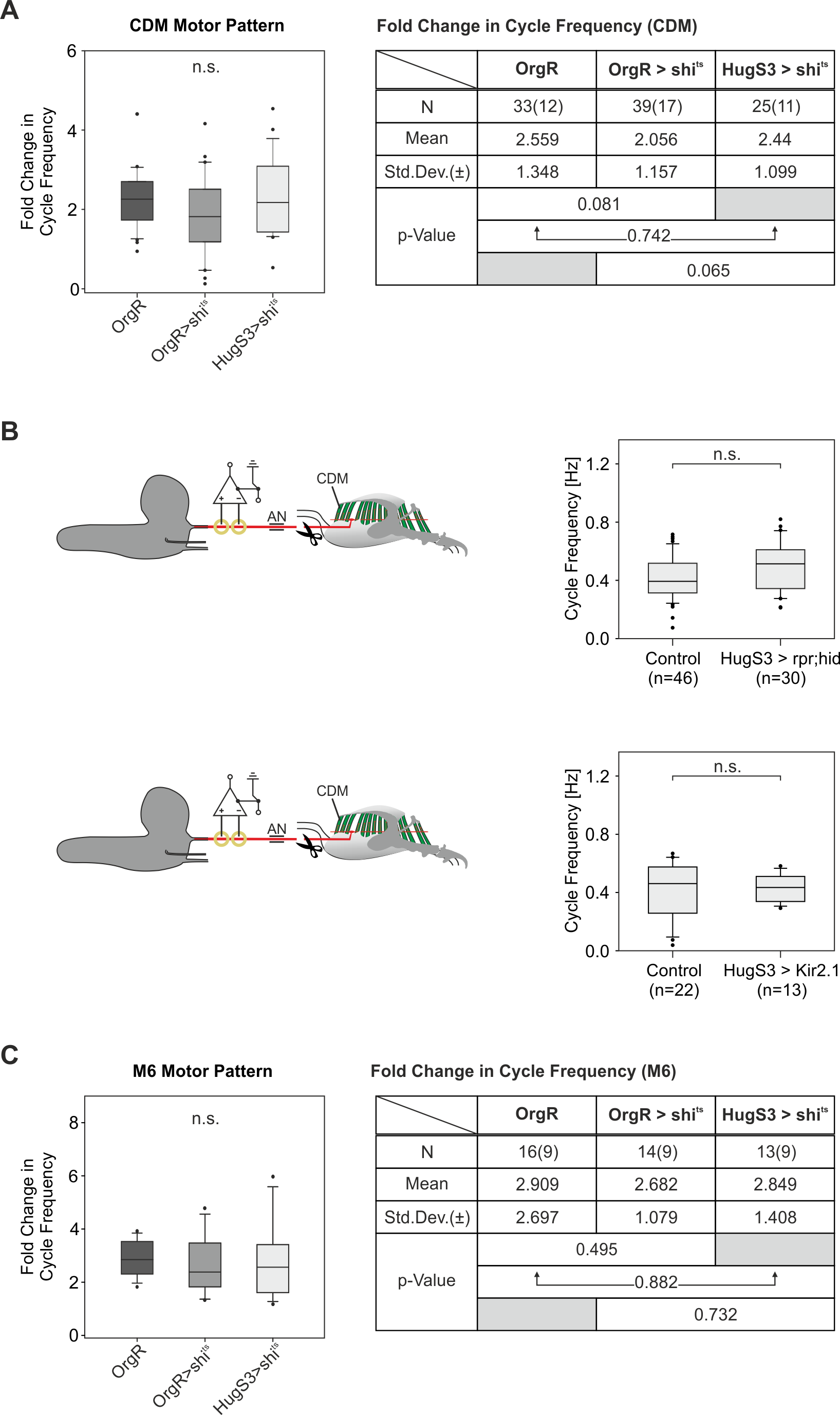

Supplement: Figure S5 — (A) Motor pattern recorded from CDM (presented as box plot for OrgR, OrgR>shits, HugS3>shits). CDM motor patterns showed no significant difference in fold change of cycle frequency between OrgR, OrgR>shits, HugS3>shits (performed Mann-Whitney Rank Sum Test (n.s. – not significant)). (B) Left side: Experimental setup for the nerve recordings of HugS3>rpr/hid (upper panel) and HugS3>Kir2.1 (lower panel). Right side: Graph shows the cycle frequency of the AN motor pattern after ablation of the hugin neurons by the apoptotic factors rpr and hid and during inhibition of hugin neurons using Kir2.1 (lower panel). Compared to the control (OrgR) inhibiting and ablating the hugin neurons showed no significant difference (performed Mann-Whitney Rank Sum Test (n.s. – not significant)). (C) Motor pattern recorded from M6 (presented as box plot for OrgR, OrgR>shits, HugS3>shits). M6 motor output showed no significant difference in fold change of cycle frequency between OrgR, OrgR>shits, HugS3>shits (performed Mann-Whitney Rank Sum Test (n.s. – not significant)). (TIF) [file pbio.1001893.s005.tif]

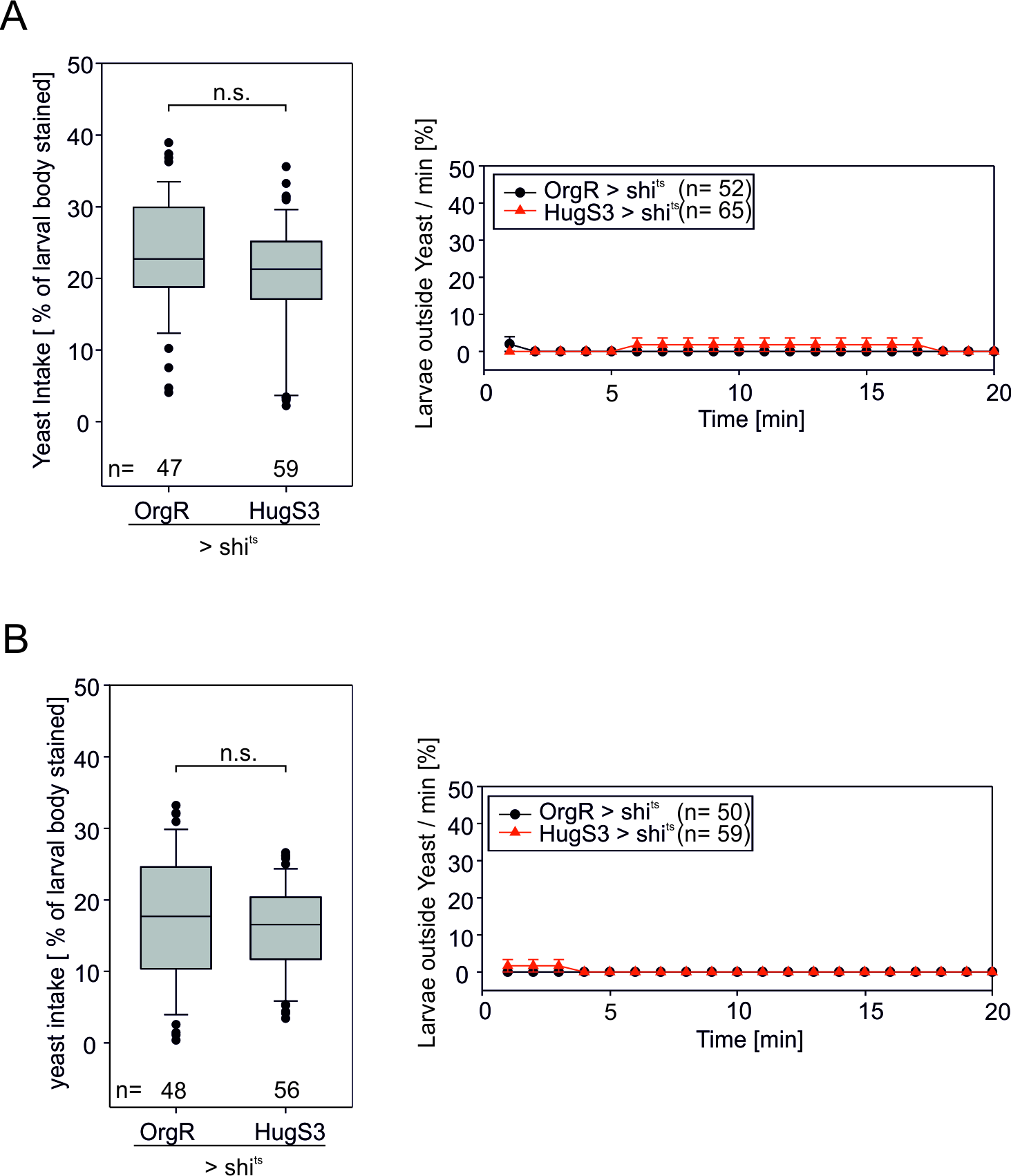

Supplement: Figure S6 — (A,B) Statistical analysis of food intake and wandering-like behavior assay of OrgR×shits compared to HugS3>shits under starved (A) and fed (B) conditions. The graph (left) shows the intake of yeast (area of the alimentary tract stained by colored yeast divided by body surface area) after 20 min at 32°C. Graph (right) illustrates the statistical data of the wandering-like behavior of OrgR×shits compared to HugS3>shits measured as larvae outside the yeast/min [%] over a time period of 20 min. In both nutritional conditions HugS3>shits showed no significant difference in food intake and wandering-like behavior relative to OrgR×shits at 32°C. (TIF) [file pbio.1001893.s006.tif]

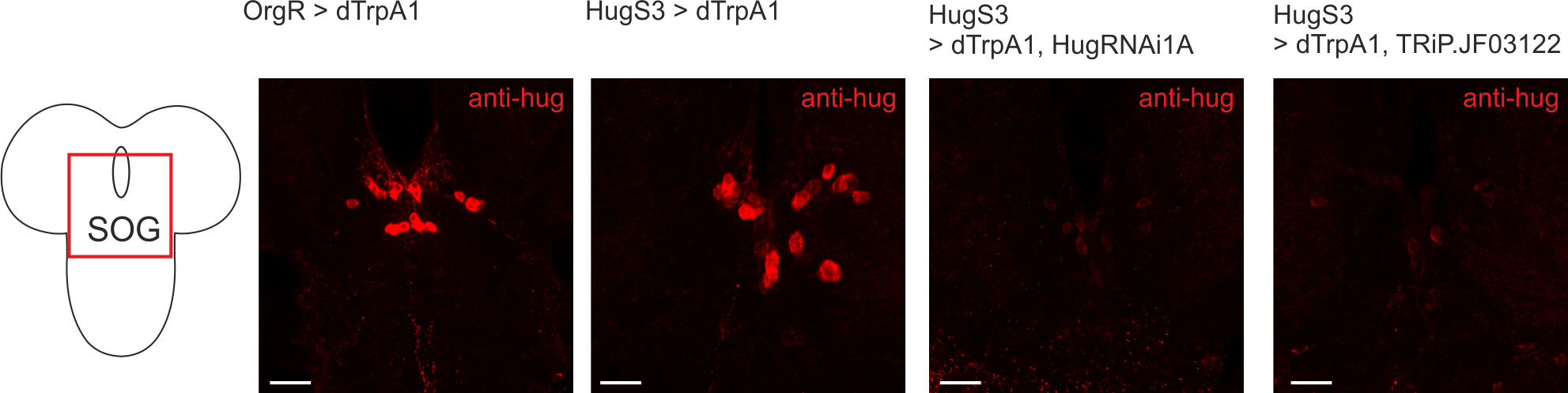

Supplement: Figure S7 — Hugin antibody staining of the genotypes: OrgR>dTrpA1, HugS3>dTrpA1, HugS3>dTrpA1, HugRNAi1A and HugS3>dTrpA1, TRiP.JF03122. Images show the subesophageal ganglion of the larval CNS as indicated in the schematic drawing (left side, scale bar: 20 µm). (TIF) [file pbio.1001893.s007.tif]

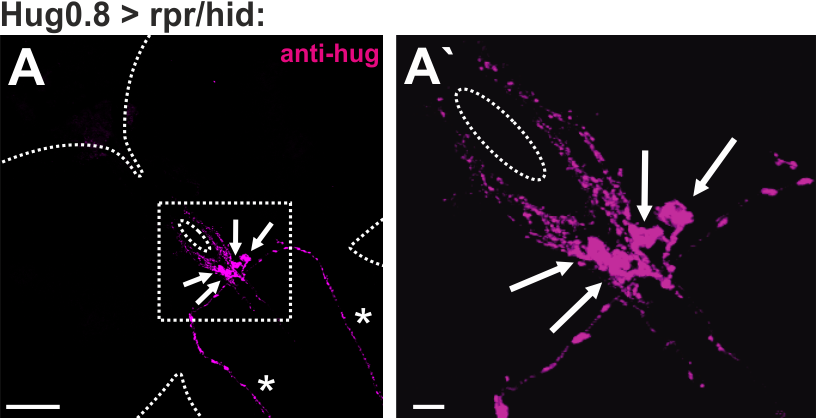

Supplement: Figure S8 — (A) Hugin antibody staining of hug0.8>rpr/hid showing four remaining cells in the SOG that project to the VNC (A, scale bar: 50 µm; A′, scale bar: 10 µm). (TIF) [file pbio.1001893.s008.tif]

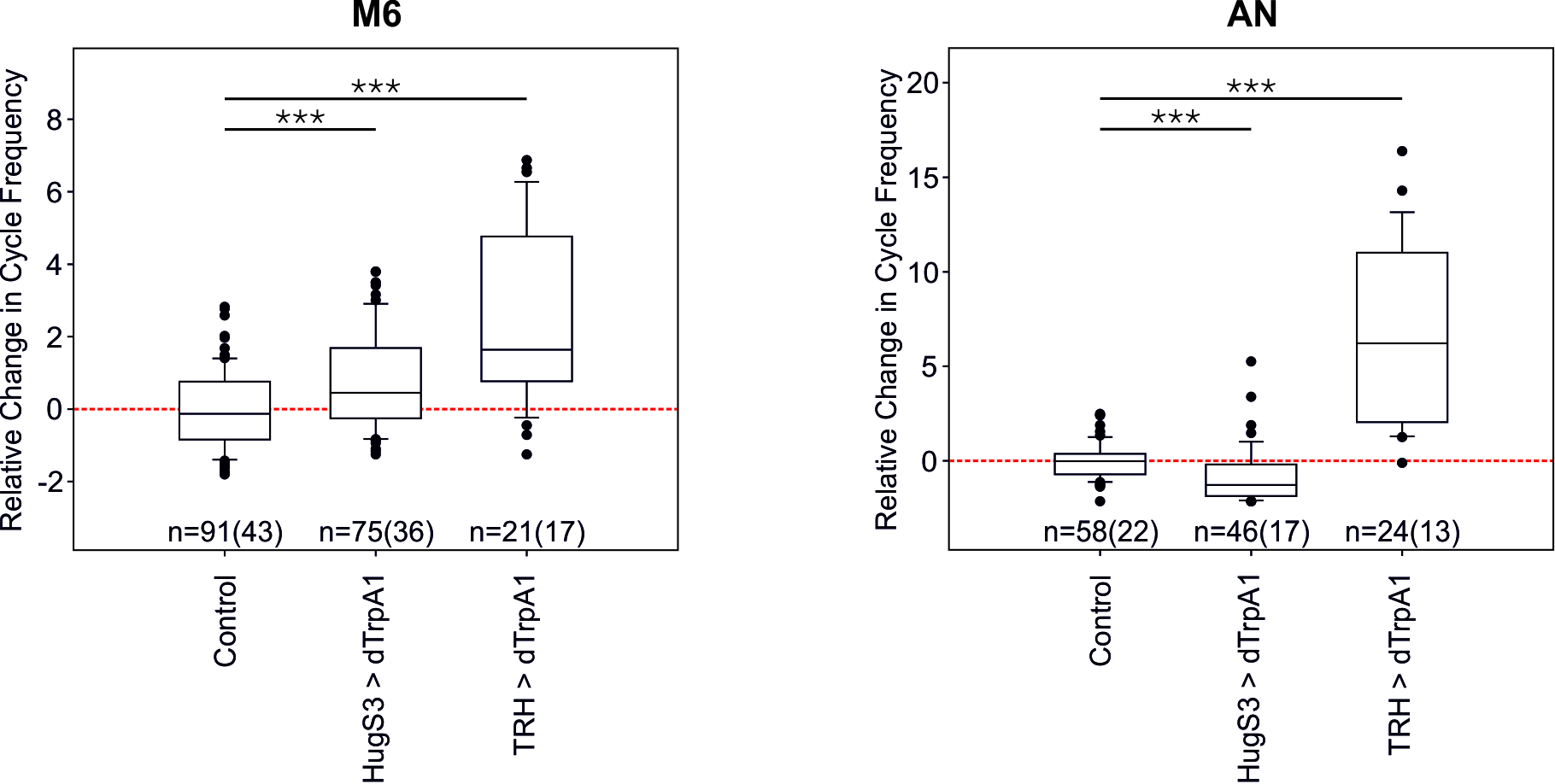

Supplement: Figure S9 — Graphs show the relative change in cycle frequency of M6- and CDM-motor pattern of HugS3>dTrpA1 and TRH>dTrpA1 compared to the control lines (Mann-Whitney Rank Sum Test: *p≤0.05, **p≤0.01, ***p≤0.001). (TIF) [file pbio.1001893.s009.tif]
