# Supplementary material for: Correction: Selection of Motor Programs for Suppressing Food Intake and Inducing Locomotion in the Drosophila Brain
Source: PLoS Biol. 2014 Nov 6;12(11):e1002016. doi: 10.1371/journal.pbio.1002016 (PMC4222583; doi:10.1371/journal.pbio.1002016)
Supplement: File S2 — Republished corrected article. (PDF) [file pbio.1002016.s002.pdf]

OPEN ACCESS Freely available online

PLOS | BIOLOGY

Selection of Motor Programs for Suppressing Food Intake and Inducing Locomotion in the *Drosophila* Brain

Andreas Schoofs[1], Sebastian Hückesfeld[1], Philipp Schlegel[1], Anton Miroschnikow[1], Marc Peters[1], Malou Zeymer[1], Roland Spieß[2], Ann-Shyn Chiang[3], Michael J. Pankratz[1]*

1 Molecular Brain Physiology and Behavior, LIMES-Institute, University of Bonn, Germany, **2** Department of Forensic Entomology, Institute of Legal Medicine, Jena University Hospital, Germany, **3** Brain Research Center, National Tsing Hua University, Taiwan

Abstract

Central mechanisms by which specific motor programs are selected to achieve meaningful behaviors are not well understood. Using electrophysiological recordings from pharyngeal nerves upon central activation of neurotransmitter-expressing cells, we show that distinct neuronal ensembles can regulate different feeding motor programs. In behavioral and electrophysiological experiments, activation of 20 neurons in the brain expressing the neuropeptide hugin, a homolog of mammalian neuromedin U, simultaneously suppressed the motor program for food intake while inducing the motor program for locomotion. Decreasing hugin neuropeptide levels in the neurons by RNAi prevented this action. Reducing the level of hugin neuronal activity alone did not have any effect on feeding or locomotion motor programs. Furthermore, use of promoter-specific constructs that labeled subsets of hugin neurons demonstrated that initiation of locomotion can be separated from modulation of its motor pattern. These results provide insights into a neural mechanism of how opposing motor programs can be selected in order to coordinate feeding and locomotive behaviors.

Citation: Schoofs A, Hückesfeld S, Schlegel P, Miroschnikow A, Peters M, et al. (2014) Selection of Motor Programs for Suppressing Food Intake and Inducing Locomotion in the *Drosophila* Brain. PLoS Biol 12(6): e1001893. doi:10.1371/journal.pbio.1001893

Academic Editor: Piali Sengupta, Brandeis University, United States of America

Received February 17, 2014; **Accepted** May 15, 2014; **Published** June 24, 2014

Copyright: © 2014 Schoofs et al. This is an open-access article distributed under the terms of the Creative Commons Attribution License, which permits unrestricted use, distribution, and reproduction in any medium, provided the original author and source are credited.

Funding: Financial support from DFG (Deutsche Forschungsgemeinschaft) grant PA787, DFG Sonderforschungsbereich SFB645 and SFB704, LIMES (Life and Medical Sciences) graduate school of Nordrhein-Westfalia (NRW), and DFG Cluster of Excellence ImmunoSensation. The funders had no role in study design, data collection and analysis, decision to publish, or preparation of the manuscript.

Competing Interests: The authors have declared that no competing interests exist.

Abbreviations: 5-HT, serotonergic; ACh, cholinergic; AEL, after egg laying; AN, antennal nerve; CDM, cibarial dilator muscles; CNS, central nervous system; CPG, central pattern generator; DA, dopaminergic; dTrpA1, transient receptor potential ion channel; Glu, glutamatergic; H, hemisphere; Hug, hugin; MN, maxillary nerve; M6, abdominal muscle M6; PaN, prothoracic accessory nerve; PSP, post-synaptic potential; RNAi, RNA interference; SOG, subesophageal ganglion; VNC, ventral nerve cord.

* Email: pankratz@uni-bonn.de

Introduction

The recruitment of appropriate motor programs to changing environmental conditions is an essential aspect of animal behavior [1]. The nervous system of invertebrates and vertebrates includes a broad variety of motor programs with neuronal circuits having the intrinsic property to generate rhythmic motor output, termed central pattern generators (CPGs). These motor programs underlie the spatial and temporal activation of specific muscle groups that characterize movements like chewing, swallowing, walking, breathing, and locomotion [2,3]. The mechanisms by which a specific motor program is selected from a repertoire of potential motor programs are not well understood.

In vertebrates, the motor system for locomotion has been extensively studied with various methods including pharmacological, electrophysiological, and more recently genetic tools [4–9]. Isolated spinal cord preparations have been used to demonstrate the existence of locomotive CPGs in the mammalian spinal cord [10,11]. The neural networks comprising the motor programs and the motor neurons for the single limbs are located in the spinal cord, and the motor network of a limb can be divided into motor subprograms and sets of motor neurons for each joint of a limb. These spinal–cortical networks are activated via reticulospinal neurons by command centers in the mesencephalon and diencephalon, which in turn are controlled by neuronal structures in basal ganglia [1,12]. Neurotransmitters such as serotonin, for example, have been shown to be necessary to induce motor patterns in isolated brainstem–spinal preparations [13,14]. Specific neurotransmitter-expressing cells that are involved in regulating the speed of locomotion have also been identified by genetic tools in lamprey, zebrafish, and mouse [15–19]. Currently, little is known about the cellular circuits in the brainstem or descending cortical pathways which regulate the locomotion CPGs in the spinal cord [20].

In addition to the highly conserved locomotor motor behaviors, those related to feeding are critical for growth and survival. These encompass movements involving the whole body for searching and getting access to food sources, local parts of the body for actual food intake, as well as organ-specific movements for post-ingestive phases of feeding. In invertebrates, the rhythmic nature of swallowing and food transport has been utilized as a model to study the structure of CPGs that generate oscillating motor patterns [3,21], as well as providing insights into the physiological parameters that drive feeding behavior [22–25]. This has also been the case in mammalian systems, where the discovery of leptin provided a major nucleation point for analyzing how peripheral signals influence central circuits that regulate food intake behavior and energy homeostasis [26,27]. Simpler genetic systems such as

Author Summary

In the animal kingdom, two of the most essential behaviors are locomotion and feeding. The motor programs underlying these behaviors are controlled by higher-order circuits in the central nervous system. However, how an organism selects a particular motor program based on inputs from the information-processing higher brain centers to generate an adaptable behavior is not well understood. Here, we analyze the behavior of *Drosophila* larvae after activating a small cluster of neurons in the brain and show that the animals simultaneously stop eating and start moving. These neurons express the neuropeptide hugin, which is homologous to the mammalian neuromedins. We show that the reduction of food intake depends on hugin and that the cluster of hugin neurons is functionally divided into distinct subgroups that both accelerate the motor program for locomotion and decelerate the motor program for feeding. We propose that hugin neurons represent a control system between the higher brain circuits that process information and those that execute motor programs.

Drosophila and *Caenorhabditis elegans* are increasingly being used to study the genes and neural circuits that control feeding and feeding-related processes. These studies include the identification of the first gene involved in food search behavior [28], metabolic genes that influence feeding, as well as numerous neuropeptide- and neurotransmitter-encoding genes, to name a few [29–32].

Studies in *Drosophila* have, to date, focused mostly on analyzing feeding behavior in response to chemosensory or metabolic cues [33–39].These studies have used sophisticated genetic tools to manipulate specific neuronal populations in the brain [40,41], but what has lagged behind is a high resolution readout of such manipulations on motor programs. Most have used behavioral paradigms as readout assays, for example extension of the proboscis towards a food source, measurement of food ingested, the direction a fly takes in two-choice food assays. Although providing valuable information, these approaches determine the summation of many motor programs, and it is difficult to deconstruct the distinct motor programs that produce the observed behavioral output. In addition, most of the feeding behavior assays are performed in response to sensory stimuli, and it is not possible to distinguish which step in the sensorimotor pathway is primarily being affected. Thus, it is not surprising that, in comparison with chemosensory circuits [42–44], much less is known about the motor circuits that underlie feeding behavior.

Recently, an electrophysiological approach was used in semi-intact preparations to monitor the rhythmic motor patterns that comprise the *Drosophila* larval feeding cycle [45]. These analyses led to the identification of three motor patterns derived from three distinct nerves that innervate the feeding apparatus and which together comprise larval feeding behavior: motor output of antennal nerve (AN) results in pharyngeal pumping, motor output of maxillary nerve (MN) drives mouth hook movements, and that of prothoracic accessory nerve (PaN) causes head tilting movements [45]. In addition to providing higher resolution dissection of feeding motor patterns, this approach also overcomes an important issue relevant for studying motor circuits in general: it eliminates external inputs provided by a wide variety of sensory organs, as well as by internal peripheral tissues that can affect feeding responses, such as the gut, fat body, or the oenocytes [46–48]. The approach provides an opportunity to combine molecular genetics with electrophysiology in order to study how the central nervous system (CNS) selects and executes motor programs.

In this study, we used behavioral, genetic, imaging, and electrophysiological approaches to study central mechanisms that modulate feeding-related behaviors. We first identified neurotransmitter and neuropeptide clusters that modulate subsets of motor programs for feeding. This revealed that a small neuronal cluster can oppositely regulate feeding and locomotive motor programs. The cells of this cluster express the gene *hugin*, which encodes a neuropeptide homolog to mammalian neuromedin U and which was previously proposed as being involved in food intake and food search behaviors [23,49]. Increased neuromedin U signaling in mammals has been shown to suppress feeding and increase locomotion [50,51]. We show here that activation of hugin neurons suppresses the motor program for feeding and simultaneously initiates the motor program for locomotion. Our results provide a model for how selection of coordinately regulated motor programs can be brought about through activation of a single cluster of neurons in the brain.

Results

Electrophysiological Analysis of Central Neurons that Alter Feeding Motor Patterns

We previously characterized the major muscles and the nerves driving the movements that underlie feeding behavior [45,47]: AN, MN, and the PaN (see also Figure 1A). Our next goal in characterizing the feeding motor system was to identify central components of the motor hierarchy that could modulate the motor pattern recorded from the three pharyngeal nerves. The strategy was to activate specific neurotransmitter- and neuropeptide-expressing neurons in an inducible manner, and assay their effect on motor programs of feeding-related behavior (Figure 1). Directing the expression of the temperature-sensitive cation channel dTrpA1 [52] via the Gal4-UAS system enabled us to characterize the effect of activating distinct neuronal populations in a temporally controlled manner (Figure S1). We initially prescreened 11 lines, representing major neurotransmitter and selected neuropeptide lines, by a food intake assay (Figure S2); those that showed significant effect on food intake were taken for electrophysiological as well as additional feeding analysis. Five lines selected for this study were those labeling glutamatergic (Glu), cholinergic (ACh), serotonergic (5-HT), dopaminergic (DA), and hugin (Hug) neurons. The effect of temperature-induced activation of neuronal populations on the motor patterns was then monitored with single extracellular recordings of the three pharyngeal nerves (AN, MN, and the PaN) to distinguish neuronal populations that would affect the feeding motor pattern either globally or as just a subset (Figure 1). We then compared the changes in cycle frequency, which is a crucial feature of rhythmic behavior: classical studies on crustacean stomatogastric nervous systems revealed that all known modulatory inputs affect the cycle frequency of pyloric motor rhythm by altering the endogenous properties of at least one component of the CPG [3].

Neuronal activation of the Glu population resulted in a reversible state of tonic excitation in the motor patterns of all three pharyngeal nerves (Figure 1B). This was expected since the Gal-4 driver line (OK371) drives target gene expression in nearly all Glu neurons of the CNS that comprise the motor neurons [53]. Activating the ACh neurons showed a significant increase in cycle frequency of all motor patterns; in some instances the pattern approached the tonic excitation seen for Glu neurons. Activation of 5-HT neurons also caused an increase in cycle frequency; the effect on the 5-HT neurons stood out because of the remarkable

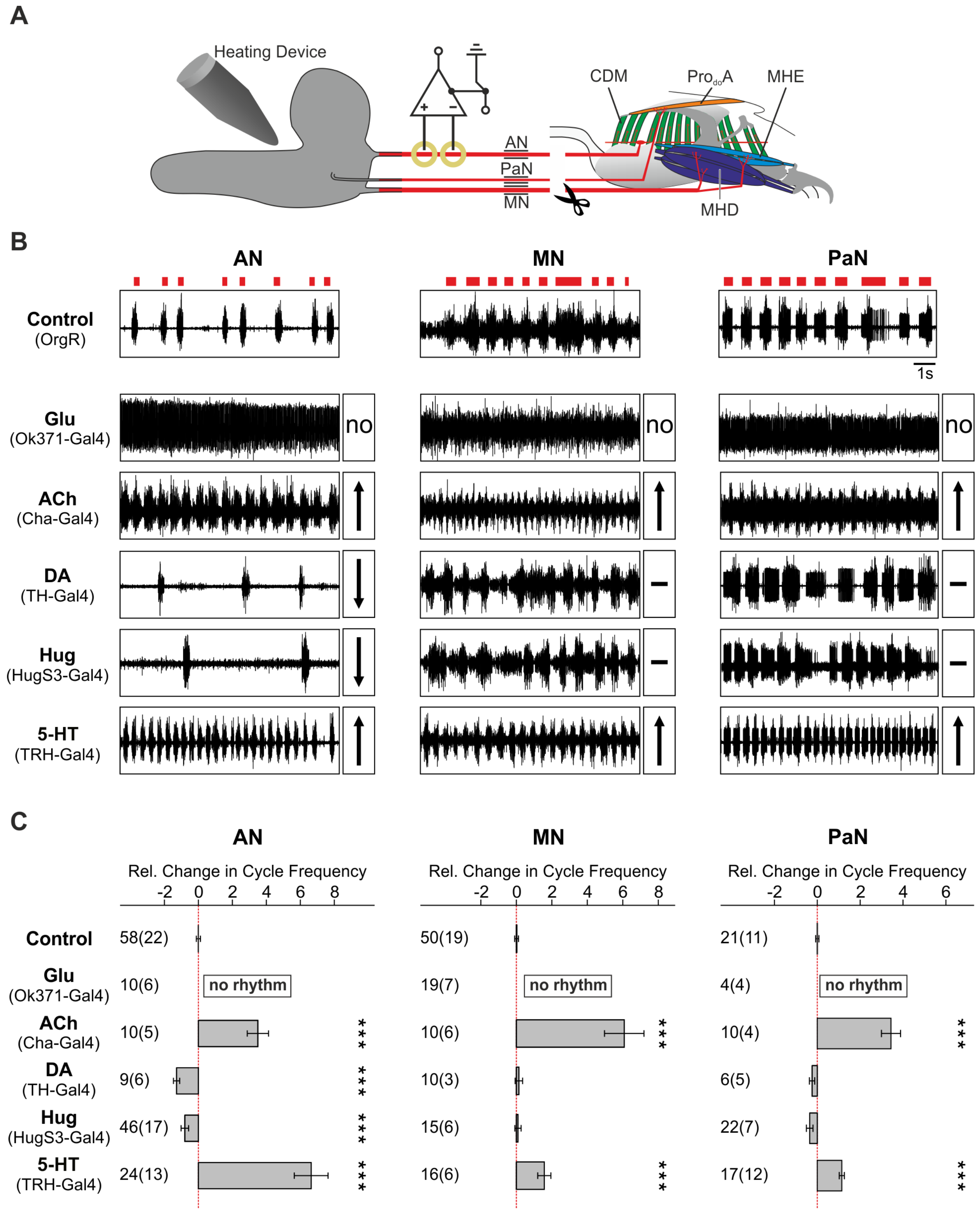

Figure 1. Identification of neuronal networks modulating motor patterns using Gal4-directed thermo-sensitive UAS-dTrpA1 expression. (A) Experimental setup for AN, MN, and PaN recordings at the deafferented CNS; dTrpA1 was activated by a Peltier-driven heating device. (B) Single extracellular recordings of AN, MN, and PaN revealed differential alteration of feeding-related motor patterns by dTrpA1 activation. Red blocks on top of the control recordings denote motor output. For the experimental recordings, an up arrow (↑) indicates significant acceleration of motor pattern, down arrow (↓) indicates significant deceleration of motor pattern and a dash (–) indicates no significant difference in the motor pattern (exception: Glu (Ok371-Gal4 showed no rhythmic motor pattern by dTrpA1 activation [no]). (C) Statistical data from AN, MN, and PaN motor patterns quantified as relative change in cycle frequency (mean ± standard error). Significance was tested by Mann-Whitney Rank Sum Test (***$p \leq 0.001$). 5-HT, serotonin; ACh, acetylcholine; DA, dopamine; Glu, glutamate; Hug, hugin neuropeptide; MHD, mouth hook depressor; MHE, mouth hook elevator; Pro$_{do}$A, dorsal protractor A.

doi:10.1371/journal.pbio.1001893.g001

regularity of the accelerated motor pattern in all three pharyngeal nerves. By contrast, activation of DA and Hug neurons decreased rhythm frequency. Moreover, these showed differential effect on the feeding motor patterns: only the AN motor pattern was affected, and not the MN or the PaN. (Figure 1B). These results indicated that certain neuronal classes affected all, whereas others affected only a subset, of the feeding motor programs.

Food intake studies further confirmed the roles of these neurones in feeding behavior. A short-term yeast intake assay was used in order to minimize longer-acting peripheral influence on the feeding response (Figure 2A). Four neuronal populations significantly decreased yeast intake: Glu, ACh, DA, and Hug neurons. Only one increased yeast intake: 5-HT (Figure 2A). Contraction of the cibarial dilator muscles (CDM), which is due to the AN motor program, is the movement most dedicated to food intake per se as compared to those driven by MN or PaN motor programs. Contractions of CDM presumably generate a negative pressure, resulting in ingestion of liquidized food: 'pharyngeal pumping'. Thus, we also performed video-based monitoring of the CDM contractions in semi-intact larvae to see how this particular movement could be correlated with the electrophysiology and food intake data (CDM tracking, Figure 2B). There is indeed a good correlation between the CDM contraction pattern and the AN recordings, which may explain the food intake results. For Glu, the tonic-like excitation resulted in convulsive contractions of the CDM, leading to essentially no food intake. For ACh, the CDM relaxed incompletely between successive contractions, causing less effective pharyngeal pumping, which likely accounts for the decreased food intake despite increased pumping rate. For DA and Hug, the frequency of the contraction was reduced (Figure 2B); the effect was more drastic for Hug, as seen by the strength of each contraction. The decreased food intake in both cases is as expected. For 5-HT, there was a rapid increase in the rate of CDM contractions, consistent with the increase in AN recording cycle frequency and food intake.

The combined electrophysiological and behavioral analyses opened up several avenues to pursue, as all the lines revealed interesting features relating to selection and modulation of motor patterns. For example, the unique finding that the serotonergic line, when activated, was the only one of 11 lines tested which resulted in increased food intake. The dopaminergic and hugin lines were interesting since they affected only a subset of the motor

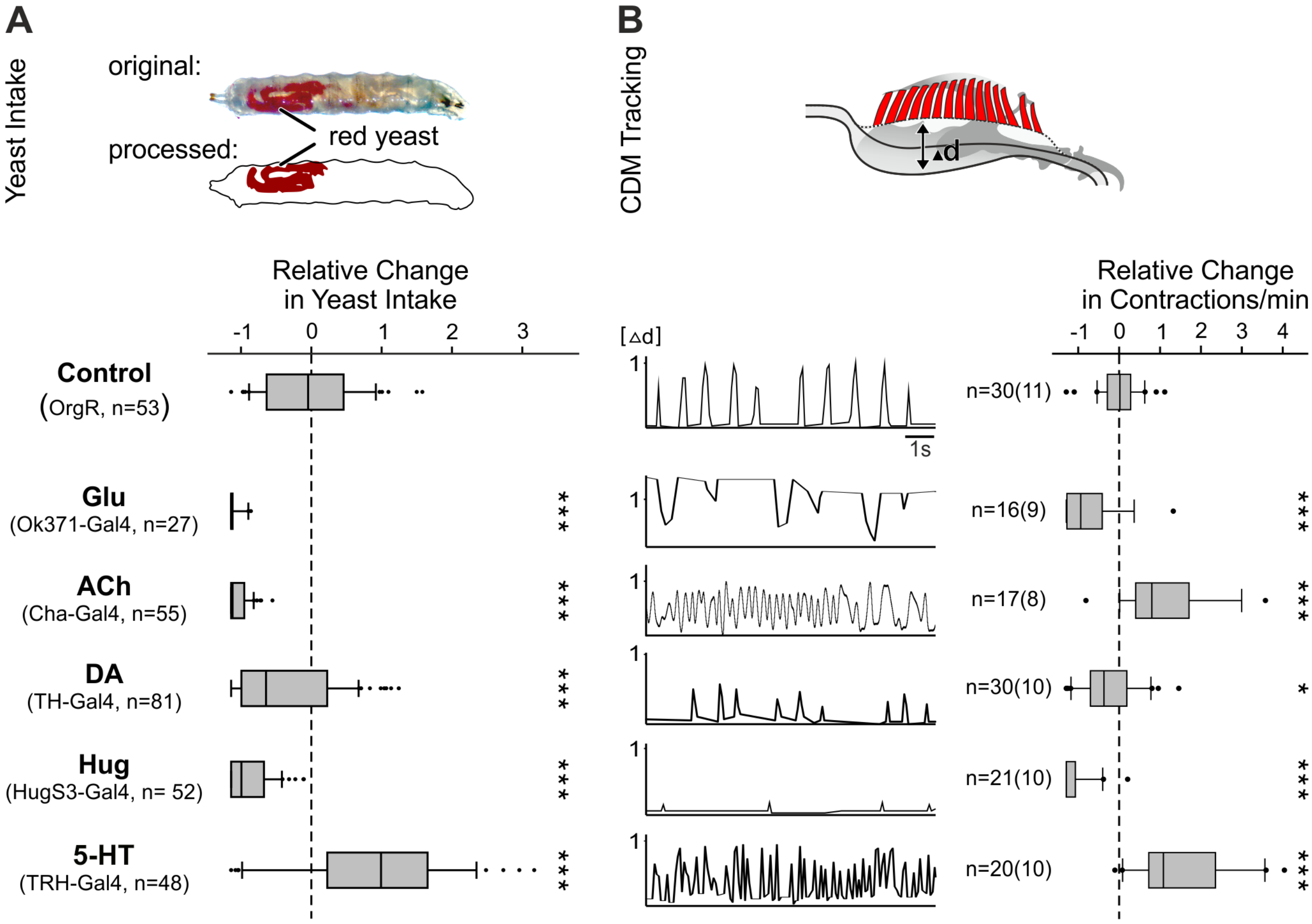

Figure 2. Effect on yeast intake and CDM contractions by Gal4-directed dTrpA1-mediated activation of neuronal networks. (A) Experimental setup: yeast intake of larvae (% of body stained) was determined after 20 min of dTrpA1 activation (upper picture). All tested Gal4-lines showed a decreased relative change in yeast intake except 5-HT (TRH-Gal4) line, which showed an increase (lower panel; Mann-Whitney Rank Sum Test: ***p≤0.001). (B) CDM contractions were tracked by measuring the length difference of pharyngeal lumen (Δd) at 32°C relative to the maximal contractions at 18°C (upper picture). Tracking of the CDM contractions correspond to deduced muscle activity based on the AN recordings (lower left panel). CDM contractions were quantified as relative change in contractions/min (lower right panel). Significance was tested by Mann-Whitney Rank Sum Test (*p≤0.05, ***p≤0.001). 5-HT, serotonin; ACh, acetylcholine; DA, dopamine; Glu, glutamate; Hug, hugin neuropeptide.
doi:10.1371/journal.pbio.1001893.g002

programs (i.e., the AN, but not MN or PaN motor programs), thus demonstrating a specificity in recruitment of different motor programs that comprise the feeding system. For the current study, we decided to focus on the hugin neuronal cluster for one critical reason, namely the relative simplicity of the expression pattern generated by the HugS3-Gal4 line in both numerical and spatial terms. Previous studies showed that this line drives reporter gene expression precisely in 20 cells, all tightly clustered in the subesophageal ganglion (SOG) [49] (Figure S3), and send projections to the ventral nerve cord and the protocerebrum, which is the higher brain center.

Activation of Neurons Expressing Hugin Neuropeptide Suppresses Feeding and Increases Wandering-like Behavior

We first wanted to verify the effect of hugin on the feeding motor system using an independent method to activate neurons. Thus, we used NaChBac and recorded the AN motor pattern [54,55]. The recordings showed a significant suppression of AN motor activity, further strengthening the view that hugin neurons suppress feeding motor patterns (Figure S4). We also wanted to perform the converse experiment by inhibiting hugin neuronal activity through the use of temperature-sensitive shibire (shibire^{ts}), which blocks synaptic transmission [56]. However, we did not observe any difference in the frequency of the AN motor pattern (Figure S5A). This indicated that activating hugin neurons suppresses AN motor activity, but inhibiting them does not increase it. We do not think this is due to the normal larval feeding motor system operating at a maximal level (since larvae are continuous feeders), since we can in fact observe an increase in motor activity when serotonergic neurons are activated. Instead, we believe that this reveals insights into the mechanism by which hugin neurons function in modulating the feeding motor system (see Discussion). Consistent with this view, ablating the hugin cells (by expressing reaper-hid to induce apoptosis [57]) or inhibiting the neuronal activity using Kir2.1 also had no effect on the AN motor pattern (Figure S5B).

Based on these observations, we next wanted to analyze the alterations in feeding behavior when hugin neurons were activated in more detail. Specifically, we wanted to determine if the suppressed food intake was accompanied by alterations in a food-related locomotory behavior, namely the wandering-like behavior. This is a behavior that is observed in certain mutant larvae which are defective in food intake, where they move away from the food source and wander about the surrounding area [47–49]. Indeed, in addition to suppression of food intake, a significant wandering-like behavior is also observed when hugin neurons are activated (Figure 3).

Due to the alteration in locomotive behavior, we next asked if the activity of the abdominal segmental muscles that underlie locomotion were affected by activating the hugin neurons. The *Drosophila* larval neuromuscular junctions of the ventral longitudinal muscle (M6 and M7) are well established and have provided valuable insight into synapse function and muscle membrane excitability [58,59]. The rhythmic motor outputs recorded from abdominal muscle M6 are representative for locomotory patterns generated by the larval CNS and likely reflect crawling behavior [60,61]. We therefore monitored the activity of the abdominal muscle M6 by intracellular recordings (Figure 4A). Interestingly,

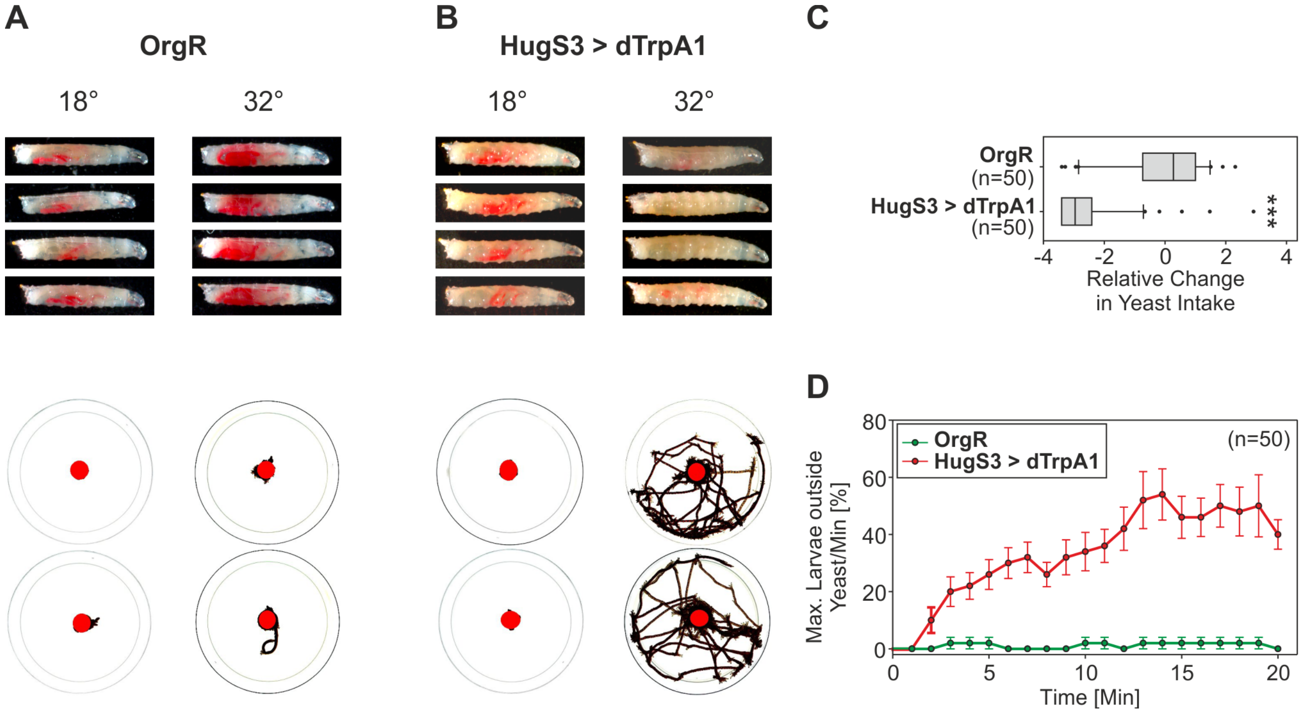

Figure 3. Behavioral consequence of dTrpA1-induced activation of hugin neurons on yeast intake and wandering-like behavior. (A–B) Photographs of OrgR (A) and HugS3>dTrpA1 (B) larvae (upper panel) and crawling tracks (lower panel) after 20 min at 18°C (no dTrpA1 activation) and 32°C (dTrpA1 activation), displaying the yeast intake and wandering-like behavior. Compared with OrgR, HugS3>dTrpA1 larvae showed reduced yeast intake and increased wandering-like behavior. (C) Activation of the hugin neurons by dTrpA1 significantly reduced the relative change in yeast intake compared with OrgR. Data is presented as a box plot (Mann-Whitney Rank Sum Test: ***$p \leq 0.001$). (D) Analysis of the locomotory activity showing that HugS3>dTrpA1 had a significantly increased wandering-like behavior (max. larvae outside the yeast/min [%]) relative to OrgR on the restrictive temperature (32°C).
doi:10.1371/journal.pbio.1001893.g003

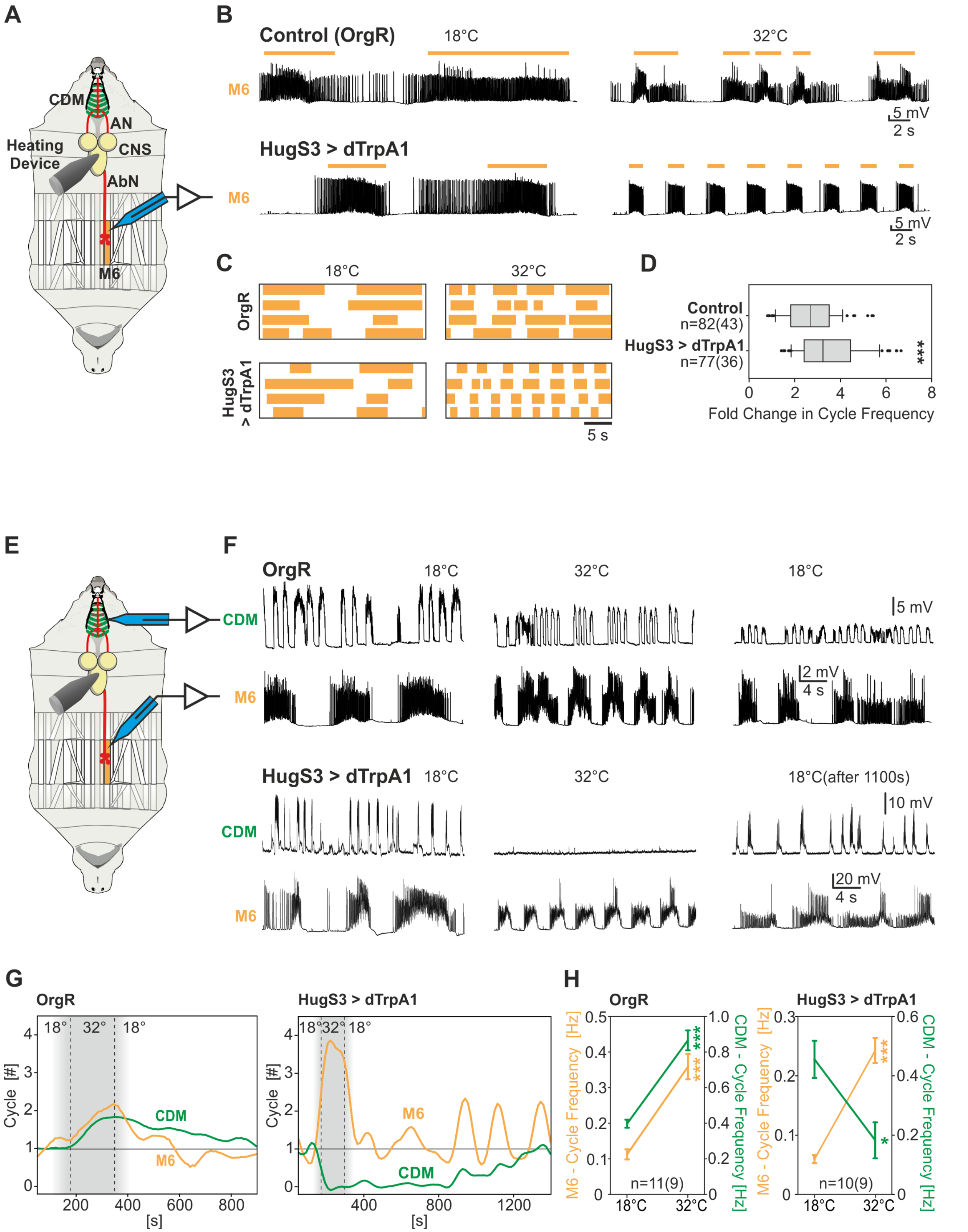

Figure 4. Hugin neurons have opposite effects on the motor patterns underlying feeding and locomotion behavior. (A) Single intracellular muscle recording of M6 (experimental setup). (B) Representative muscle recordings of OrgR and HugS3>dTrpA1 at 18°C (before dTrpA1 activation) and 32°C (during dTrpA1 activation); activation of the hugin neurons leads to an acceleration of the M6 motor pattern (colored bars

indicate bursts of PSPs). (C) Increased acceleration effect of dTrpA1 induced activation of the hugin neurons on the motor pattern (indicated by colored bars) for individual muscle recordings. (D) Activation of the hugin neurons significantly increased cycle frequency (presented as box plot) of the M6 motor pattern (Mann-Whitney Rank Sum Test: $***p \leq 0.001$). (E) Double intracellular muscle recording of the CDM and M6 (experimental setup). (F) Representative CDM/M6 recordings of OrgR and HugS3>dTrpA1 at 18°C (before dTrpA1 activation), at 32°C (during dTrpA1 activation), and after shift down to 18°C. Note the opposite effect on the CDM and M6 motor patterns at 32°C. (G) Temporal progression of CDM and M6 motor activity for OrgR- and HugS3>dTrpA1 recordings (F) upon temperature stimulation. The graph shows the number of cycles per bin (bin size: 20 s) over the recording. (H) Temperature shift from 18°C to 32°C increased the cycle frequency of the CDM and M6 motor pattern of OrgR in the same manner, whereas in the case of HugS3>dTrpA1 the CDM cycle frequency decreased and the M6 cycle frequency increased (symbols indicate the mean, whiskers indicate the standard error). Significance was tested by Mann-Whitney Rank Sum Test ($*p \leq 0.05$, $***p \leq 0.001$). AbN, abdominal nerve.
doi:10.1371/journal.pbio.1001893.g004

we observed an accelerating effect on abdominal muscle contraction pattern upon activation of the hugin neurons (Figure 4B–D). We note at this point that we also recorded the M6 muscle motor pattern when shibire^{ts} was used to silence hugin neurons, but as with the pharyngeal motor pattern above, no effect was observed (Figure S5C).

We then asked if pharyngeal pumping and abdominal activity could be coordinately regulated. Therefore, we performed double intracellular recordings of the CDM and the abdominal muscle M6 (Figure 4E). Remarkably, hugin neuron activation resulted in a concomitant decrease in feeding and increase in locomotion motor program: the post-synaptic potentials (PSPs) of CDM are completely suppressed, whereas those of abdominal muscle (M6) persist and the motor pattern increased in cycle frequency (Figure 4F–H). In wild type situations, an increase in temperature results in the usually observed temperature effect where both activities are increased (Figure 4H). It is well established that temperature has a profound effect on intrinsic network properties that influence the setting of rhythm frequencies in the CNS of invertebrates and vertebrates [62,63]. In HugS3>dTrpA1, the increased abdominal motor activity is accompanied by a concomitant decrease in CDM motor activity. These results indicated that hugin neurons can modulate two opposite motor programs simultaneously: the feeding program and the locomotory program. Consistent with the previous results, shibire^{ts} also had no effect on the CDM motor pattern recordings (Figure S5A) and the underlying feeding behavior (Figure S6).

Hugin Neuropeptide Is Required to Suppress the Feeding Motor Program

Since the results described above indicated that activation of the hugin neurons leads to suppression of feeding, we next wanted to determine if the hugin neuropeptide is required for this suppression. The strategy was to decrease hugin neuropeptide levels in the hugin neurons through RNA interference (RNAi) and see if activating the hugin neurons would still result in suppression of feeding behavior. First we determined the effectiveness of several RNAi lines to decrease hugin neuropeptide levels (Figure 5A; Figure S7). We chose two independent constructs that were effective in reducing hugin neuropeptide levels (HugRNAi1A and Hug-TriPJF03122).

Animals which only expressed the *hugin* RNAi gene construct did not show any alterations in the feeding phenotype, in line with the results, described in the previous section, showing that inhibiting or ablating hugin neurons also had no effect (Figure S5). However, if hugin neurons were activated with dTrpA1 in animals expressing the HugRNAi construct, the suppression of AN motor pattern was no longer observed (Figure 5B, top panel). Similar results were observed with food intake and wandering-like behavior. In both cases, the HugRNAi lines significantly prevented the hugin neurons from exerting their suppressive effect (Figure 5C). Interestingly, the increase in cycle frequency of M6 motor pattern was not affected—that is, activating the hugin neurons still resulted in increased cycle frequency (Figure 5B, bottom panel). Thus, the induction of wandering-like behavior can be decoupled from modulation of the locomotory motor program. Taken together, these results show that hugin neuropeptide is required for modulating food intake but not for the locomotion motor program; it is also required for initiating wandering-like behavior.

Distinct Cells of the Hugin Cluster Modulate Speed of Abdominal Muscle Contraction

The hugin neuronal cluster comprises just 20 cells, whose soma are all located in the SOG. Earlier work showed that the hugin neurons form four distinct subclasses, each having different projection targets [23,49]. One subclass sends projections down the entire length of the ventral nerve cord (VNC) [64], suggesting a possible role in locomotion. To explore this, we made several deletion constructs of the hugin cis-regulatory region in order to see if the different subclasses were under the control of separable enhancers. In one construct (Hug0.8) there was a complete absence of expression in the four hugin cells that project to the VNC (Figure 6A–C; G), whereas the other 16 neurons were present. Furthermore, using this promoter element in cell ablation experiments resulted in the loss of the 16 cells, whereas the four hugin VNC neurons remained (Figure S8), demonstrating the specificity of this promoter element. To analyze the behavioral consequence, we carried out both food-intake and wandering-like locomotion assays. The 16-cell construct (Hug0.8), in which the VNC projections were missing, could still suppress food ingestion as well as induce wandering-like behavior (Figure 6H,I).

We then performed the converse experiment: to determine the function of the 4-cell hugin cluster that projects to the VNC. We therefore made a promoter construct from a region that was deleted in Hug0.8 construct relative to the HugS3 construct. This line drove target gene expression in precisely the four hugin cells that project down the VNC (Figure 6D–F, G). dTrpA1 activation of this 4-cell VNC cluster had no effect on food intake or wandering-like behavior (Figure 6 H,I).

Next we measured cycle frequency of the AN motor pattern after dTrpA1 activation of these two nonoverlapping neuronal clusters. The hugin-0.8 line suppressed the AN motor pattern, whereas the VNC-line could not (Figure 7A–C), supporting the food intake data. However, when M6 abdominal muscle recordings were performed, we observed the acceleration of the motor pattern with the 4-cell element but not with the 16-cell element (Figure 7D–F). Similar results were obtained when we performed simultaneous double recordings from CDM (for pharyngeal pumping) and M6 abdominal muscles (Figure 7 G–I).

Taken together, these results indicated that food intake (motor program for pharyngeal pumping) and initiation of wandering-like behavior can be decoupled from modulation of the speed of abdominal muscle contraction. The 4-cell hugin VNC cluster can thus regulate locomotion speed separately from pharyngeal pumping. Therefore, although activation of the entire 20-cell hugin cluster coordinately suppresses feeding and enhances locomotion speed, the two motor programs are under the control of distinct hugin neuronal subclasses. Both the suppression of food intake and the induction of wandering-like behavior are performed by the 16-cell cluster, whereas the 4-cell VNC cluster is required to increase the cycle frequency of the locomotor motor pattern.

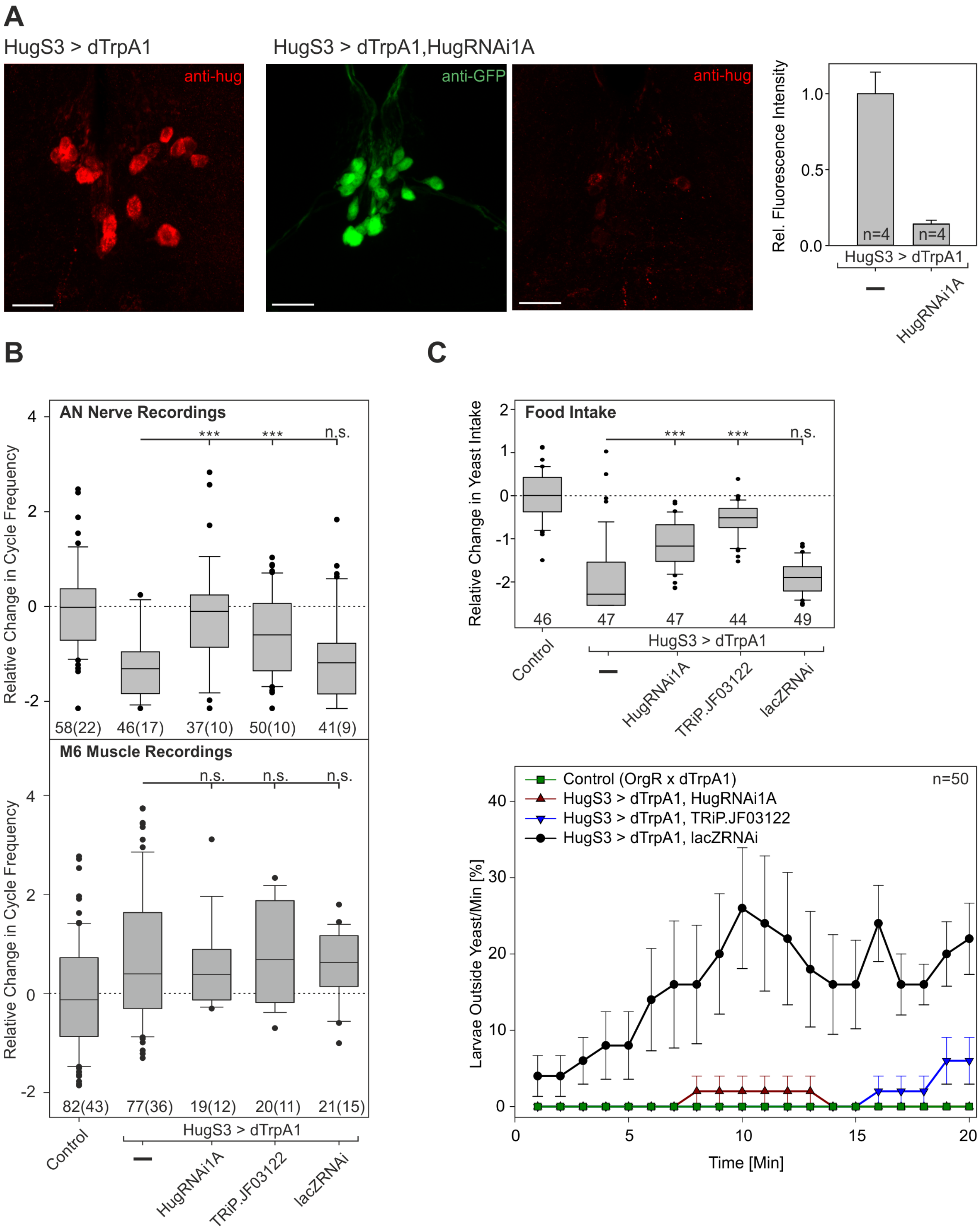

Figure 5. Analysis of hugin neuropeptide function in feeding and locomotion by *hugin* RNAi. (A) Antibody staining of CNS from HugS3>dTrpA1 larva with hugin antibody (left panel). Double staining of CNS from HugS3>dTrpA1,HugRNAi1A larva (middle two panels); this *hugin* RNAi construct also expresses GFP (scale bar: 20 µm). Fluorescence intensity analysis of hugin antibody staining indicates significant decrease of hugin neuropeptide for HugS3>dTrpA1,HugRNAi1A compared with HugS3>dTrpA1 (antibody staining of all genotypes is shown in Figure S7). LacZRNAi serves as control RNAi construct. (B) Analysis of AN motor pattern was quantified as relative change in cycle frequency (upper panel). Recordings

revealed that HugS3>dTrpA1,HugRNAi1A showed a complete, and HugS3>dTrpA1,TRiP.JF03122 a partial, rescue by the RNAi on the motor output most dedicated to food ingestion. Analysis of M6 muscle recording results (lower panel) is presented as relative change in cycle frequency). HugS3>dTrpA1,HugRNAi1A and HugS3>dTrpA1,TRiP.JF03122 showed a significant difference compared with the control and no significant difference to the HugS3>dTrpA1. In contrast to AN motor pattern and wandering-like behavior, the effect of HugS3>dTrpA1 on motor pattern of muscle M6 could not be rescued by the knock down of the hugin neuropeptide (see text for discussion, Mann-Whitney Rank Sum Test: n.s., nonsignificant; ***p≤0.001). (C) Analysis of food intake behavior (upper panel). Results are presented as relative change in yeast intake. HugS3>dTrpA1,HugRNAi1A and HugS3>dTrpA1,TRiP.JF03122 showed a significant difference to control and HugS3>dTrpA1, indicating partial rescue by two independent RNAi constructs. Analysis of locomotor activity is presented as larvae outside the yeast/min at 32°C (during dTrpA1 activation) over a time period of 20 min (lower panel). Knock down of hugin neuropeptide in the two *hugin* RNAi harboring animals prevented induction of wandering-like behavior; the effect is similar to Control (OrgR, OrgR x dTrpA1), and significantly different to HugS3>dTrpA1,lacZRNAi (Mann-Whitney Rank Sum Test: n.s., nonsignificant; ***p≤0.001).
doi:10.1371/journal.pbio.1001893.g005

The Protocerebrum Is Required for Suppression of the AN Motor Pattern

The above results indicated that the 16 cell hugin cluster mediates the suppressive effect of hugin neurons on the AN motor pattern. These comprise three different subclasses of hugin neurons [49,64]: two of these have projections which leave the CNS and target the periphery (to the pharynx, and the ring gland), and one has projections to the protocerebrum. In an effort to start addressing the issue of whether the protocerebrum is required for hugin function in modulating feeding motor pattern, we used a

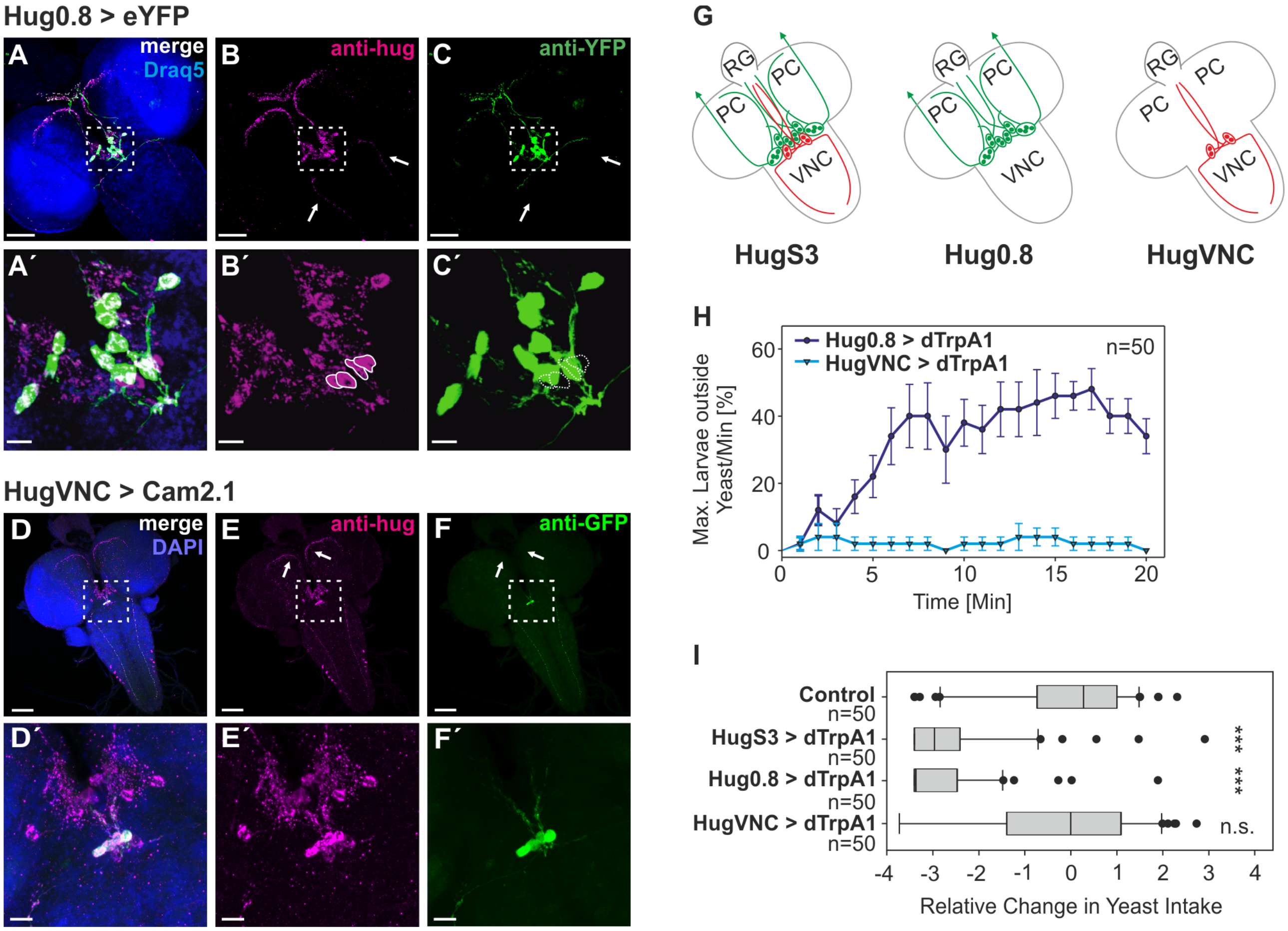

Figure 6. Effect of different subclasses of hugin neurons on the motor pattern underlying feeding and locomotion behavior. (A–C) Double antibody staining of Hug0.8: fluorescence expression driven by Hug0.8-Gal4 (C). Cell bodies and aborizations labelled by hugin antibody (B); merge of B and C (A). (A′–C′) Magnification of labeled somata in the SOG (magnified region indicated by dashed box in the original image (A–C)). Hug0.8 lacks the four hugin cells (marked in B′ and C′) which project to the VNC (indicated by arrows in B and C). (D–F) Double antibody staining of HugVNC: fluorescence expression driven by HugVNC-Gal4 (F). Cell bodies and aborizations labelled by hugin antibody (E); merge of E and F (D). (D′–F′) Magnification of labeled somata in the SOG (magnified region indicated by dashed box in the original image (D–F)). Only the four cells that project to the VNC are labelled. Arrows mark the missing projections to protocerebrum (A–F: 50 µm, A′–F′: 10 µm). (G) Schematic summary of the three different hugin promoter constructs. HugS3 drives target gene expression in all 20 hugin cells; Hug0.8 lacks the four cells that project to the VNC; HugVNC drives expression only in the four cells that project to the VNC. (H) At activating temperature (32°C), HugVNC>dTrpA1 animals displayed no wandering-like behavior whereas Hug0.8>dTrpA1 animals displayed increased wandering-like behavior similar to HugS3>dTrpA1 (see Figure 3D). Locomotor activity was measured as max. larvae outside the yeast/min [%]. (I) Relative change in yeast intake after 20 min of dTrpA1 activation. Control (OrgR), HugS3>dTrpA1, Hug0.8>dTrpA1, and HugVNC>dTrpA1 animals were measured for food intake after 20 min of dTrpA1 activation (32°C). In comparison with the control, HugS3>dTrpA1 and Hug0.8>dTrpA1 showed a significant decrease in food intake (Mann-Whitney Rank Sum Test: n.s., nonsignificant; ***p≤0.001).
doi:10.1371/journal.pbio.1001893.g006

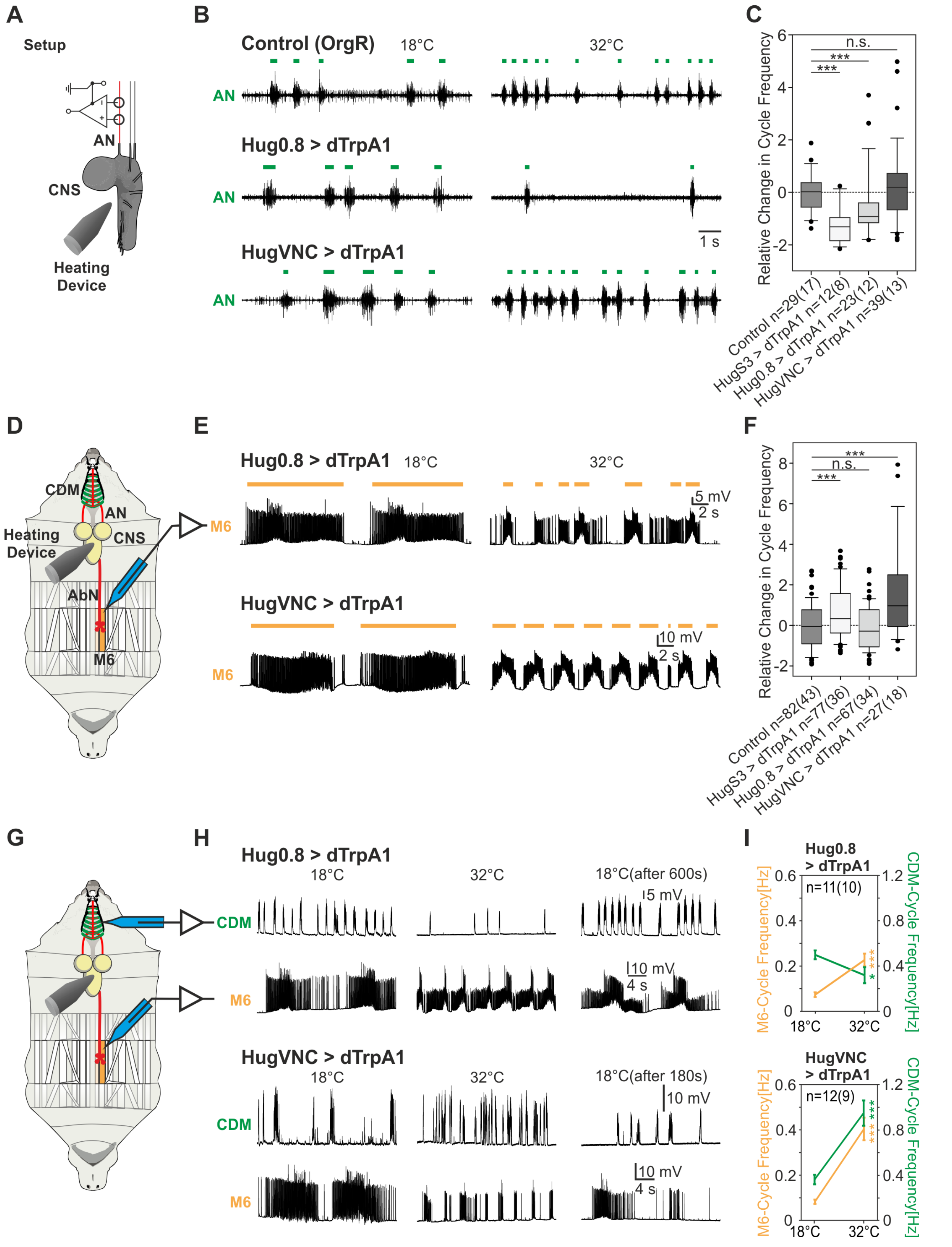
A
Setup
AN
CNS
Heating Device
B
Control (OrgR)
18°C
32°C
AN
Hug0.8 > dTrpA1
AN
1 s
HugVNC > dTrpA1
AN
C
Relative Change in Cycle Frequency
n.s.
***
***
Control n=29(17)
HugS3 > dTrpA1 n=12(8)
Hug0.8 > dTrpA1 n=23(12)
HugVNC > dTrpA1 n=39(13)
D
CDM
AN
Heating Device
CNS
AbN
M6
E
Hug0.8 > dTrpA1
18°C
32°C
M6
5 mV
2 s
HugVNC > dTrpA1
10 mV
2 s
M6
F
Relative Change in Cycle Frequency
***
n.s.
***
Control n=82(43)
HugS3 > dTrpA1 n=77(36)
Hug0.8 > dTrpA1 n=67(34)
HugVNC > dTrpA1 n=27(18)
G
H
Hug0.8 > dTrpA1
18°C
32°C
18°C(after 600s)
5 mV
CDM
M6
10 mV
4 s
HugVNC > dTrpA1
18°C
32°C
18°C(after 180s)
10 mV
CDM
10 mV
4 s
M6
I
Hug0.8 > dTrpA1
n=11(10)
M6-Cycle Frequency[Hz]
CDM-Cycle Frequency[Hz]
18°C
32°C
HugVNC > dTrpA1
n=12(9)
M6-Cycle Frequency[Hz]
CDM-Cycle Frequency[Hz]
18°C
32°C

Figure 7. Effect of different subclasses of hugin neurons on the motor pattern underlying feeding and locomotion behavior. (A) Experimental setup of AN recording for dTrpA1 activation. (B) Representative AN recordings of control (OrgR), Hug0.8>dTrpA1, and HugVNC>dTrpA1 at 18°C (before dTrpA1 activation) and 32°C (during dTrpA1 activation). Activation of dTrpA1 in Hug0.8-Gal4 significantly decreased the cycle frequency of the AN-motor pattern, but not in HugVNC-Gal4 (colored bars indicate the motor pattern). (C) Relative change in cycle frequency of the AN-motor pattern by dTrpA1 activation in control, HugS3>dTrpA1, Hug0.8>dTrpA1, and HugVNC>dTrpA1, illustrated as box plots (Mann-Whitney Rank Sum Test: n.s., nonsignificant; ***p≤0.001). The effect of 20-cell hugin cluster on the CDM motor pattern was verified by a second genetic tool to activate neurons (tubGal80^{ts}; NaChBac; for details see Figure S4). (D) Experimental setup of abdominal muscle M6 recordings. (E) Representative M6 recordings of Hug0.8>dTrpA1 and HugVNC>dTrpA1 showing the motor patterns (colored bars) at 18°C (before dTrpA1 activation) and 32°C (during dTrpA1 activation). (F) Analysis of M6 motor pattern revealed a significant increase (Mann-Whitney Rank Sum Test: n.s., nonsignificant; ***p≤0.001) in relative change in cycle frequency (presented as box plot) by dTrpA1 activation for HugVNC, similar to HugS3. (G) Double intracellular muscle recording of the CDM and M6 (experimental setup). (H) Representative CDM/M6 recordings of Hug0.8>dTrpA1 and HugVNC>dTrpA1 at 18°C (before dTrpA1 activation), at 32°C (during dTrpA1 activation) and after shift down to 18°C. Hug0.8>dTrpA1 affected only the CDM motor pattern and HugVNC>dTrpA1 only the M6 motor pattern at 32°C. (I) Temperature shift from 18° to 32°C decreased the cycle frequency of Hug0.8>dTrpA1 for the CDM but not M6 motor pattern, which was comparable to OrgR (see Figure 4). For HugVNC>dTrpA1 the CDM cycle frequency increased as in OrgR, M6 cycle frequency increased (symbols indicate the mean, whiskers indicate the standard error, Mann-Whitney Rank Sum Test: *p≤0.05; ***p≤0.001).
doi:10.1371/journal.pbio.1001893.g007

classical lesion approach in combination with dTrpA1 activation. The experimental strategy was to make lesions to the isolated CNS preparation and record the AN motor pattern upon dTrpA1 activation of hugin neurons (Figure 8).

At 18°C, when dTrpA1 is not activated, lesioning the VNC or the brain hemispheres (H) still resulted in a rhythmic motor pattern from the AN (Figure 8, 18°C), although there were some noticeable variations relative to the pattern generated by an intact CNS. Upon dTrpA1 activation, the suppression of AN motor pattern was still observed when the VNC was lesioned (Figure 8B). However, when the hemispheres were lesioned, we no longer observed this suppression (Figure 8C). These results suggested that the protocerebrum is required for hugin neuronal function in suppressing the AN motor pattern underlying pharyngeal pumping. Furthermore, these results demonstrate that the CPG for the AN motor pattern is located in the SOG.

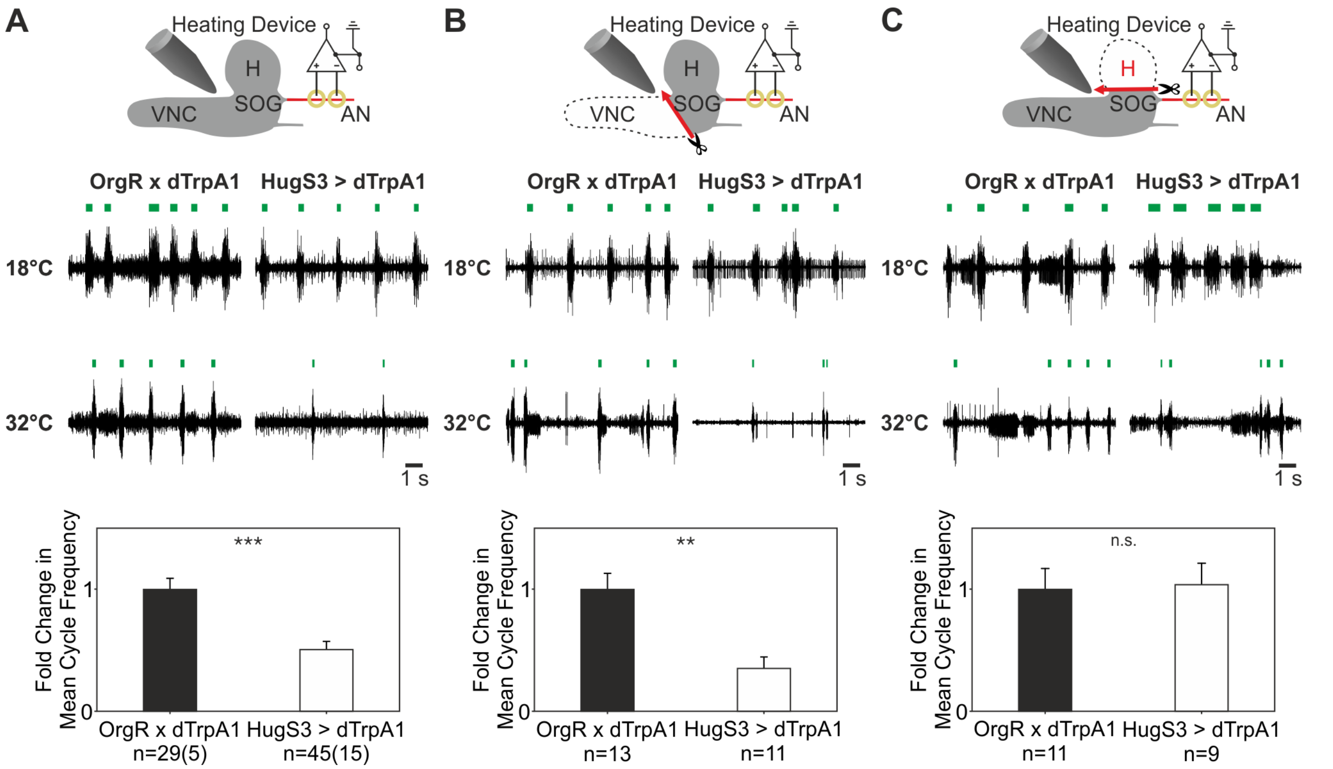

Figure 8. Lesion experiments of OrgR>dTrpA1 and HugS3>dTrpA1. (A) AN recording of the intact CNS (experimental setup, upper panel). At 18°C, OrgR×dTrpA1 and HugS3>dTrpA1 show a rhythmic motor output. At 32°C (dTrpA1 activation), the motor pattern of HugS3>dTrpA1 is decelerated (middle recordings). Analysis of the AN motor pattern during dTrpA1 activation of both genotypes quantified as fold change in mean cycle frequency (lower panel). (B) AN recording after removal of VNC (experimental setup, upper panel). Representative AN recording of OrgR×dTrpA1 and HugS3>dTrpA1 at 18°C and 32°C (dTrpA1 activation). During dTrpA1 activation, the deceleration of motor pattern effected by HugS3>dTrpA1 was still observed after removing the VNC (middle recordings). Analysis of the AN motor pattern during dTrpA1 activation of both genotypes quantified as fold change in mean cycle frequency (lower panel). (C) AN recording after removal of the brain hemispheres (experimental setup, upper panel). In HugS3>dTrpA1, lesion of the brain hemispheres resulted in no deceleration of the AN motor pattern during dTrpA1 activation (representative AN recordings of both genotypes; middle recordings). Analysis of the AN motor pattern during dTrpA1 activation of both genotypes quantified as fold change in mean cycle frequency (lower panel: Mann-Whitney Rank Sum Test: n.s, nonsignificant; **p≤0.01, ***p≤0.001).
doi:10.1371/journal.pbio.1001893.g008

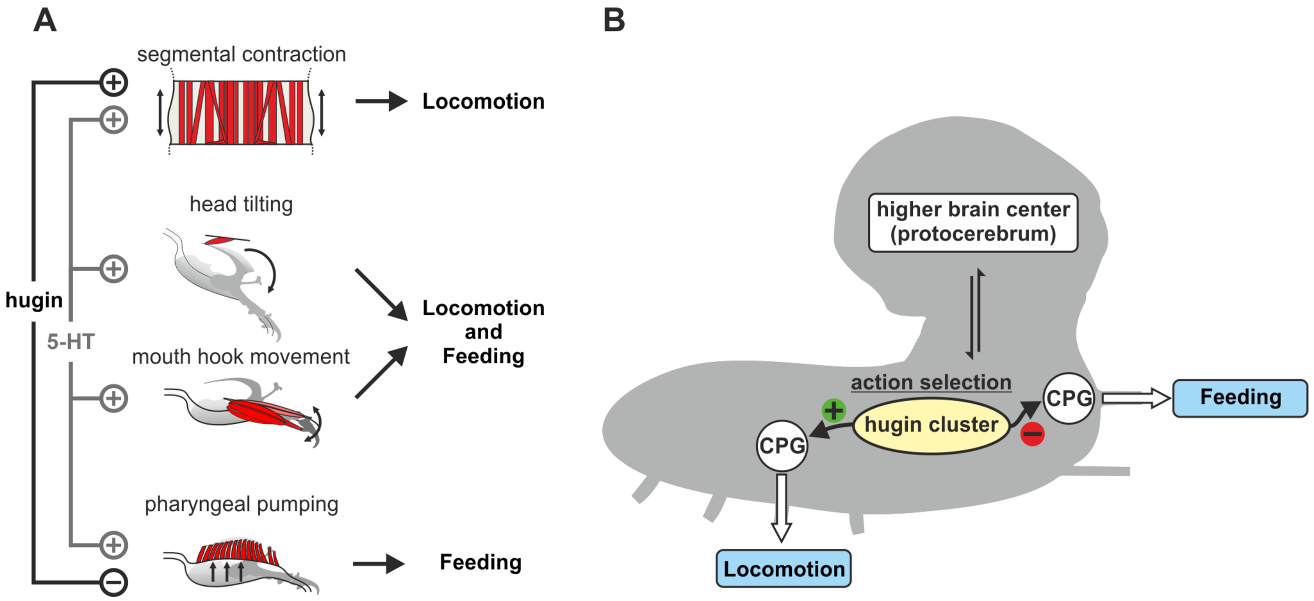

Figure 9. Model for the selection of motor programs. (A) Illustration of effect of neuronal populations on different motor programs. Hugin neurons affect a subset (pharyngeal pumping), whereas serotonergic neurons affect all feeding motor patterns (head tilting, mouth hook movement, and pharyngeal pumping). Hugin neurons also regulate in an opposite manner the motor program for segmental contraction, whereas serotonergic neurons affect segmental contraction in the same manner as the feeding motor pattern. (B) Activation of the 20-cell hugin cluster simultaneously suppresses feeding and initiates locomotion motor programs (see text for details). 5-HT, serotonergic neurons.
doi:10.1371/journal.pbio.1001893.g009

Discussion

Action Selection of Motor Programs for Different Behavioral Modules

Behavioral modules can be seen to be composed of distinct motor programs that are differentially recruited based on adaptive needs [1]. These can be cooperative or antagonistic, and the right combinations must be selected in order to bring about the required behavior. For feeding, this requires motor programs that allow actual ingestion of food as well as those locomotor programs involved in food search or food avoidance behaviors. This implies that animals must distinguish motor programs that run serially or in parallel, and those that are essentially mutually exclusive. In humans for example, feeding normally requires arm movements to bring the food to the mouth, followed by biting and chewing, and finally swallowing; but the act of swallowing can occur in the absence of the earlier movements; conversely, similar arm movements to those made during eating can be observed during running. Within a given motor program there are additional levels of modulation—for example, the speed with which a given movement is made.

The behavioral module that comprises *Drosophila* larval feeding is also composed of distinct motor programs, as shown by motor patterns of three pharyngeal nerves, the AN, MN, and the PaN [45]. Our electrophysiology screen reveals distinct populations of central neurons that can regulate motor patterns in a different manner. Some, such as serotonergic neurons, affect all three motor programs; others, like hugin neurons, act on a subset. These differences can be viewed as having varying degrees of functional overlap. Pharyngeal pumping (due to the AN motor program) is the movement most dedicated to food intake; at the other end of the functional spectrum, the segmental longitudinal muscle contractions would be most dedicated to locomotion. Mouth hook and head tilt movements (due to MN and PaN motor programs, respectively) are likely involved in both feeding and locomotion. These motor programs can be separately regulated and recruited for different behavioral modules. Such mechanisms have been demonstrated at the CPG level in other invertebrates, where the same neurons can be used in different CPGs [21,65].

For both feeding and locomotion, the cellular identities of the CPGs remain largely unknown. Previous studies have demonstrated the existence of feeding CPG(s) in the *Drosophila* larval CNS [45]. We have now localized one of these, the AN motor pattern underlying pharyngeal pumping, to the SOG by lesion experiments. Spieß et al. [66] has provided evidence that the motor neurons of the AN are also located in the SOG. Although the lesioning of VNC or brain hemisphere can still generate a rhythmic motor pattern, it is not identical to that generated when both are present, indicating that inputs from the VNC and brain hemispheres have a modulatory effect on the pharyngeal pumping CPG. Our results on feeding are complementary to earlier findings on the motor program for locomotion. Forward crawling of *Drosophila* larvae is composed of repetitive wave-like contractions of the segmental body wall musculature from posterior to anterior [67]. Several studies indicated that the neural networks of the crawling motor program (CPGs for crawling) are located in the thoracic and abdominal segments of the central nervous system [60,68], and genetic manipulations showed that the brain hemispheres and the SOG are not required to produce a rhythmic motor pattern in the VNC and crawling behavior, although the rhythmic motor pattern is required for directed movements in response to chemosensory cues [68].

In this context, a major issue is that of cellular specificity: which of the cells targeted by these neurotransmitter Gal4 lines are responsible for the observed effects on the feeding and locomotor program? For example, serotonin is expressed in about 84 cells in the larval CNS: ~56 in the VNC, ~8 in the SOG, and ~20 in the protocerebrum [69]. As mentioned above, the activation of the serotonergic cells results in an increased cycle frequency of all three motor programs; it also enhances the locomotion program in the VNC (see Figure S9, Figure 9A). However, we do not know

which of the serotonergic cells contribute to which of these programs. In addition, different groups of serotonergic cells may have different, even opposite functions, and it may be that the promoting effect dominates when all groups become activated. The various sparse lines and intersectional strategies to narrow down the types of cells being manipulated will be valuable in addressing this issue. This can be combined with the ability to record, from isolated CNS, both feeding and locomotor motor patterns, permitting the identification of central neurons that coordinate the motor programs underlying different behavioral modules.

Neuropeptide Modulation of Mutually Exclusive Actions

A striking finding from our study is the fact that activating a small cluster of 20 neurons in the SOG, all expressing the neuropeptide hugin, leads to a simultaneous suppression of a motor program for feeding and induction of one for locomotion. This is observed both at the behavioral and electrophysiological level. Thus, the hugin cluster can regulate two essentially competing programs since larvae, as with most animals, do not feed and move at the same time. A notable feature of the hugin neuronal cluster is that we have not been able to observe any difference to the control situation when hugin neuronal activity is decreased. For both pharyngeal pumping and wandering-like behavior, it is only when the hugin neurons are activated that we see a modulatory effect. Similarly, the increase in the frequency of M6 abdominal muscle contraction is observed only under activation of hugin neurons. We believe these observations provide insights into the mechanism by which the hugin neurons act. This can be illustrated in terms of how a brake and gas pedal function to coordinate two mutually exclusive operations of a car. Activating hugin neurons decreases feeding, but inhibiting them does not increase feeding: applying a brake causes deceleration, but removing it does not cause acceleration. Similarly, activating the hugin neurons enhance abdominal muscle contraction, but their inhibition does not slow down contraction: stepping on the gas pedal increases speed, but taking it off does not actively decrease speed. This scenario can be used to explain the requirement of hugin neuropeptide in our RNAi experiments. Lowering the level of hugin neuropeptide in activated hugin neurons no longer affected the motor patterns underlying food intake and locomotion, indicating that hugin neuropeptide is necessary for the hugin neurons to suppress feeding and induce wandering-like behavior.

It is of interest to note that hugin neuropeptide does not seem to be required for speeding up the motor program for locomotion. This could be because of the residual quantity of hugin neuropeptide or to some compensation mechanism; more likely, the accelerating effect is due to a different neurotransmitter. At this point, we do not know which classical neurotransmitters are expressed in the hugin cells. In mammals, it has been shown that serotonergic and cholinergic systems influence the speed of motor neuron firing in the spinal cord that underlies locomotion [6,10]. Furthermore, our results show that modulation of the speed of locomotion motor program can be decoupled with the initiation of wandering-like behavior. The decision to both stop feeding and to move out of the food is mediated by a separate cluster of 16 hugin cells, eight of which project to the protocerebrum. A possible scenario is that the cells which adjust the speed of locomotion are recruited during or after the selection of motor programs for suppressing food intake and initiating wandering-like behavior.

Neural Substrate of Action Selection: the Protocerebrum–SOG Corridor

In many vertebrates, the center for swallowing is thought to be localized in the brainstem [70–72]. The cranial nerves that innervate muscles involved in chewing and swallowing descend from the brainstem. The neuronal components are much less understood relative to the spinal cord, although identifying the specific brain areas that regulate food intake is a focus of intense study in the mouse [73]. In *Drosophila*, the larval SOG occupies a central position within the CNS to integrate information on feeding and locomotion, as it connects the VNC with the brain hemispheres. The pharyngeal nerves that innervate the feeding musculature originate from the SOG, and gustatory sensory neurons send projections to the SOG [43,45]. The brainstem in vertebrates is analogously positioned, being located at the junction between the brain and the spinal cord, and the cranial nerves that innervate the pharynx originate from this part of the CNS [74], suggesting that the SOG could represent an analogous structure to the vertebrate brainstem.

It has recently been postulated that the insect central complex might play an analogous role to the basal ganglia [75–77]. Although a canonical central complex has not yet been identified in the *Drosophila* larval brain, a functional analogue is probably located in the protocerebrum. The neuroanatomy of the hugin neurons, especially exemplified by the projection pattern that connects the SOG to the protocerebrum, suggests that the SOG/protocerebrum corridor encompassing the hugin neuronal projections may play an important role in action selection of motor programs underlying feeding and locomotion (Figure 9B). The hugin projections to the protocerebrum and the connections to the gustatory cells and the insulin-producing cells [49,78], would process external and internal sensory cues, and determine which motor programs modulating feeding and locomotion are selected.

A major future challenge will be to determine how the different neuronal components of the feeding motor hierarchy are interconnected. One essential effort will be to analyze the receptor for the hugin neuropeptides. Two putative receptors have already been identified and it would be necessary to determine the cells that express the receptors [79,80]. Another effort will be to localize the classical neurotransmitters that may be expressed in the different hugin cells. Complementary to these would be to exploit the high-resolution connectivity mapping of the larval CNS that is currently being done through serial EM reconstructions [81], as has been done in the classic work for *C. elegans* [82]. Working on a small brain with its limited behavioral repertoire may thus lead to a functional map superimposed on the connectome of the larval motor system.

Materials and Methods

Flies

The following Gal4 driver and UAS effector lines were used: OK371-Gal4 (Bloomington #26160), Cha-Gal4 (Bloomington #6798), GAD-Gal4 [83], TRH-Gal4 [84], TH-Gal4 (Bloomington #8848), DDC-Gal4 (Bloomington #8849), TDC2-GaL4 (Bloomington #9313), DILP2-Gal4 [85], HugS3-Gal4 [49], NPF-Gal4 (Bloomington #25682), sNPF-Gal4 (Kyoto DGRC #113901 (NP6301)), UAS-dTrpA1 (Bloomington #26263), UAS-eYFP (Bloomington #6659), UAS-mCD8-mRFP (Bloomington #27398), 10×UAS-mCD8-GFP (Bloomington #32184), UAS-LacZRNAi (a gift from M. Jünger), UAS-shibire^{ts} (a gift from A. Thum), and UAS-TRiP.JF03122 (Bloomington#28705). Stable homozygous lines of tubulin-Gal80^{ts} and UAS-NaChBac (a gift from R. Jackson (Tufts University)) and of tubulin-Gal80^{ts} (Bloomington #7108) and UAS-eYFP (Bloomington #6660) as control for NaChBac experiments were used. For control experiments OregonR (wild type) or w^{1118} was used.

Adult flies and larvae were reared on standard fly food and kept at 25°C unless otherwise stated. All experiments were performed with third instar larvae 98±2 h AEL (after egg laying). Four hours egg collections were made on apple juice-agar plates with yeast-water paste. After 48 h, larvae were transferred into vials containing standard fly food. For experiments with shibire^{ts} larvae were raised at 18°C to avoid temperature-induced developmental defects [56]. Experiments were performed with third instar larvae 8 days old. In the experiments using tubulin-Gal80^{ts} and UAS-NaChBac/UAS-eYFP larvae were raised on 18°C for 7 days and were transferred on 30°C for 8–12 h prior to the experiment to induce the expression of the NaChBac/eYFP.

Generation of Transgenic hug0.8-Gal4 and hugVNC-Gal4 Line

For Hug0.8-Gal4 line, a 793 bp hugin promoter fragment was amplified by primer1: CATTGACATTGCCCCCATT and primer2: GGGACAACTGATGCCAGC, subcloned into TOPO TA pCRII vector (Invitrogen), digested with BamHI and NotI and ligated into the pCasperAUG-Gal4-X vector (Addgene plasmid 8378, [86]). The construct was injected into w$^{[1118]}$ (Bloomington#3605). For HugVNC-Gal4, a 403 bp hugin promoter fragment was amplified by primer1: ATCGCAGTGCTCACAATCTG and primer2: GTGGGGCATCCTGTTTAATG from wild type DNA and subcloned into TOPO TA pCRII vector. A BamHI/NotI digestion product was ligated into pENTR4 Gateway Entry vector (Invitrogen) and cloned into the destination vector pBPGUw (Addgene plasmid 17575), [87], by using LR Clonase II enzyme mix (Invitrogen). Transgenic lines were generated using standard methods for PhiC31 integrase-mediated genomic integration into y,w; P{CaryP}attP2 (BestGene Inc, USA).

Electrophysiology

Reduced semi-intact preparations were made of third instar larva consisting of the CNS, CPS, and associated pharyngeal nerves and muscles. Detailed description of the dissection has been described earlier [45]. All dissections and experiments were performed in saline solution composed of (in mM): 140 NaCl, 3 KCl, 2 $CaCl_2$, 4 $MgCl_2$, 10 sucrose, and 5 HEPES [88].

For *en passant* extracellular recording, the nerve was insulated with a surrounding petroleum jelly border on a piece of Parafilm. Recording electrodes were made of silver wire (diameter: 25–125 µm, Goodfellow). Motor output was measured by differential recordings of the deafferented nerve with a preamplifier connected to a four-channel amplifier/signal conditioner (Model MA 102/103; Ansgar Büschges group electronics lab). All recorded signals were amplified (amplification factor: 5000) and filtered (bandpass: 0.1–3 kHz). The recordings were sampled at 20 kHz. Data was acquired with Micro3 1401 or Power 1401 mk2 A/D board (Cambridge Electronic Design) and Spike2 software (Cambridge Electronic Design).

For intracellular muscle recordings, semi-intact CDM/M6 preparations of third instar larvae were used. PSPs of the muscle M6 of 4th abdominal segment and CDM were recorded using glass microelectrodes filled with 3 M KCl solution (tip resistance: 20–30 MΩ) connected to an intracellular amplifier (BRAMP-01R, npi electronic GmbH). All recordings were digitally sampled by a Micro3 1401 or Power 1401 mk2 A/D board (Cambridge Electronic Design) at 20 kHz. Data was acquired with Spike2 software (Cambridge Electronic Design).

For analysis, data pairs of successive 60 s or 120 s recording-sections under unstimulated and stimulated conditions were analyzed. Processing of the electrophysiological recordings was performed with a modified script of Spike2 (provided by Cambrigde Electronic Design). For a pair of successive recording-sections, fold change in cycle frequency was calculated. The dTrpA1-experiments revealed an endogenous temperature effect which could mask the impact of dTrpA1-activated GAL4-driver lines on the rhythmic motor output. Due to this, the mean fold change in cycle frequency of the respective control experiments was subtracted for each data point, termed relative change in cycle frequency.

Temperature Stimulation

For dTrpA1-experiments (nerve/muscle recording and CDM tracking) thermal stimuli were applied to the dorsal side of CNS. The custom-made stimulator consisted of a silver wire (diameter: 4 mm) attached to a Peltier element with thermally conductive adhesive. Peltier element was driven by a voltage-regulated power supply (VSP 2405, Voltcraft) connected to an A/D board. The end of the thermal stimulator was filed to a tip and insulated with nail polish. Applied temperature was measured by digital thermometer (GMH 3210, Greisinger electronic). The sensor for the thermometer was placed 5 mm from the tip (for temperature calibration see Figure S1). Temperature signals were acquired with the A/D board. The thermal stimulator was regulated by a script-based feedback loop via the A/D-board.

Behavioral Assay

For measurement of yeast ingestion, larvae were first washed and then starved in a Petri dish lined with tap water-moistened tissue for 30 min on RT. Afterwards they were transferred on colored yeast (colored with crimson red powder) on pre-warmed (30 min at 32°C) apple juice-agar plates and incubated for 20 min at 32°C. Afterwards the larvae were removed from the yeast and placed in 65°C hot water. For analysis larvae were photographed using a digital camera (Axiocam ICc 1, Zeiss) mounted on a binocular (Stemi 2000-CS, Zeiss). For each individual, the amount of yeast ingested was calculated as area of the alimentary tract stained by colored yeast divided by body surface area using the software ImageJ (Fiji). Data on the feeding assay is represented as percentage of ingested yeast relative to the body surface.

For simultaneous investigation of feeding and wandering-like behavior, five larvae were placed on a pre-heated/-cooled apple juice agar plate (18°C or 32°C). 20 min videos at 18°C and 32°C were acquired using a digital camera (Quickcam 9000 Pro, Logitech) and the software VirtualDub. The measurement of yeast ingestion was performed as listed in the previous paragraph. The locomotion data was analyzed using the tracking software MTrack2 (Fiji). Analysis of larvae leaving the yeast spot was carried out using a custom-made macro for ImageJ (Fiji).

Monitoring CDM Activity

CDM contractions were studied in semi-intact larvae. The preparation consisted of the CNS, the abdominal body wall, and the feeding apparatus (CPS including associated muscles). Thermal stimulation was applied directly to the CNS. Consecutive videos of 60 s at 18°C and 60 s at 32°C were recorded using a digital camera (Axiocam ICc 1, Zeiss) mounted on a binocular (Stemi 2000-CS, Zeiss). CDM contractions were tracked by measuring the length-difference of pharyngeal lumen (Δd) over time relative to the maximal contractions at 18°C. The measurements were performed using the software ImageJ (Fiji).

Immunohistochemistry

Dissected larval brains were fixed in paraformaldehyde (4%). For the antibody staining of hug-eYFP, primary antibody was rabbit-antiGFP (1:500, Abcam plc) and the secondary antibody

was rabbit-antiGFP Cy2 (1:200, Dianova GmbH). The antibody staining of HugVNC>Cam2.1 was performed with chicken anti-GFP (1:500, Abcam plc) and as secondary antibody anti-chicken Alexa488 (1:200, Invitrogen) was used. Antibody staining of hugin was performed with guinea pig anti-Hugin (1:200, Pankratz laboratory; for hug0.8>rpr/hid) or rabbit anti-Hugin (1:500, Pankratz laboratory; hug0.8>eYFP). Antibody stainings for RNAi experiments were done using rabbit anti-Hugin (1:500).Secondary antibodies were: anti-rabbit Cy3, anti-guinea pig Cy3 (1:200, Jackson ImmunoResearch), and mouse anti-GFP (1:500, Sigma-Aldrich). Nuclei were counter stained with DAPI or Draq5. Labeled larval brains were mounted in Mowiol. Imaging was carried out using Laser Scanning Microscope (ZEISS LSM780). The obtained images were arranged using Zen LE and Photoshop CS5 (Adobe) (for detailed staining procedures see [64]).

Fluorescence Microscopy

All images were obtained by using a confocal microscope Zeiss LSM 780; non-specific background fluorescence of the in vivo images was reduced by the Median Filter of the Zeiss Zen Software.

Cloning of *Hugin* RNAi Construct

Hugin cDNA PCR fragment flanked by a *Bam*HI and a *Kpn*I restriction sites was cloned into *pHIBS* vector [89] (primer sequences GGATCCGTTCCATTCGATCGTCCGAC and GGTACCGTGGCACTGGCCTTCTGG). The 394 bp hugin fragment represents bases 41 to 434 of 1033 bp hugin full length cDNA (flybase.org). A 478 bp *Sal*I/*Kpn*I fragment of hugin-*HIBS* was then cloned into *Xho*I/*Kpn*I cut *pUdsGFP* [89]. Next a 407 bp *Bam*HI/*Eco*RI fragment of hugin-*HIBS* was cloned into the *Eco*RI/*Bgl*II cut hugin-*pUdsGFP*. The *pUdsGFP* plasmid harboring two hugin fragments in opposite orientation was used for standard germline transformation [90]. The line used in the text is referred to as HugRNAi1A.

Lesion Experiments

For the lesion experiments we used the standard reduced semi-intact preparations of third instar larvae as mentioned above (see Electrophysiology). VNC or brain hemispheres were removed by a microdissecting scissor (Fine Science Tools). Five minutes after the lesion of the neuronal tissue, extracellular recording of antennal nerve was started. Thermal stimuli were applied by the above described protocol for temperature stimulation. Consecutive 60 s sections of the AN motor output at 18°C and 32°C were analyzed. The cycle frequency of AN motor pattern at 18°C showed no significant difference between OrgR×dTrpA1 and HugS3>dTrpA1 for each lesion. Therefore the data is presented as fold change in cycle frequency of AN motor pattern between both genotypes at 32°C (during dTrpA1 activation) for each experiment.

Data Analysis

All electrophysiological and behavioral experiments were tested for significance with the Mann-Whitney-Rank-Sum-test (*$p \leq 0.05$, **$p \leq 0.01$, ***$p \leq 0.001$).

Supporting Information

Figure S1 (A) Experimental set up of heating device calibration. (B) Calibration curve of the heating device (x-axis – $T_{heat\ element}$ [°C], y-axis – $T_{environment}$ [°C]). At $T_{heat\ element}$ 18°C the measured $T_{environment}$ was 19.8+/−0.48°C and at $T_{heat\ element}$ 32°C the measured $T_{environment}$ was 26.9+/−0.3°C.
(TIF)

Figure S2 (A) Experimental setup: yeast intake of larvae [% of body stained] was determined after 20 min of dTrpA1-activation. The following major neurotransmitter systems were used for the initial screening: glutamatergic (Glu), cholinergic (ACh), GABAergic (GABA), serotonergic (5-HT), dopaminergic (DA), combined serotonergic/dopaminergic (5-HT/DA) and combined octopaminergic/tyraminergic (OA/TYR) neuronal populations. We also tested four neuropeptide genes shown in earlier studies to be involved in some aspect of feeding response: *Drosophila* insulin-like peptide (Dilp), hugin (Hug), neuropeptide F (NPF) and short NPF (sNPF) (see Materials and Methods for the respective Gal4-lines). (B) Statistical data of yeast intake screen for all tested Gal4-lines is represented as box plots. Crosses were categorized based on their effect on larval food intake (Mann-Whitney Rank Sum Test: *$p \leq 0.05$, **$p \leq 0.01$, ***$p \leq 0.001$).
(TIF)

Figure S3 (A–C) Antibody staining of HugS3>10×GFP expression pattern in the CNS; Magnification (A′–C) of hugin cell cluster (20 cells) in the SOG (A: scale bar: 50 µm; A′: scale bar: 10 µm). Schematic summary of the projection pattern HugS3-Gal4 line in the larval CNS (right side). Target region of the projections are: PC, RG, SOG, VNC and periphery via PaN. Abbr.: CNS – central nervous system; PaN – prothoracic accessory nerve; PC – protocerebrum; RG – ring gland; SOG – subesophageal ganglion; VNC – ventral nerve cord.
(TIF)

Figure S4 (A) Experimental set up of AN recordings at the isolated CNS. Larvae of both genotypes were 166+/−2 h old (raised on 18°C) and kept for at least 8–12 h on 30°C before recording. (B) Original AN recordings of HugS3>tubGal80^{ts};eYFP and HugS3>tubGal80^{ts};NaChBac (colored boxes represent the CDM activity). (C) Box plot of the cycle frequency [Hz] of HugS3>tubGal80^{ts};eYFP (mean (std. dev.): 0.423 (+/−0.121); number of larvae (number of experiments): 29(10)) and HugS3>tubGal80^{ts};-NaChBac (mean (std. dev.): 0.196 (+/−0.192); number of larvae (number of experiments): 30(10)). HugS3>tubGal80^{ts};NaChBac was significant different to HugS3>tubGal80^{ts};eYFP (p-value≤0.001). Abbr.: AN – antennal nerve; CDM – cibarial dilator muscle; CNS – central nervous system.
(TIF)

Figure S5 (A) Motor pattern recorded from CDM (presented as box plot for OrgR, OrgR>shi^{ts}, HugS3>shi^{ts}). CDM motor patterns showed no significant difference in fold change of cycle frequency between OrgR, OrgR>shi^{ts}, HugS3>shi^{ts} (performed Mann-Whitney Rank Sum Test (n.s. – not significant)). (B) Left side: Experimental setup for the nerve recordings of HugS3>rpr/hid (upper panel) and HugS3>Kir2.1 (lower panel). Right side: Graph shows the cycle frequency of the AN motor pattern after ablation of the hugin neurons by the apoptotic factors rpr and hid and during inhibition of hugin neurons using Kir2.1 (lower panel). Compared to the control (OrgR) inhibiting and ablating the hugin neurons showed no significant difference (performed Mann-Whitney Rank Sum Test (n.s. – not significant)). (C) Motor pattern recorded from M6 (presented as box plot for OrgR, OrgR>shi^{ts}, HugS3>shi^{ts}). M6 motor output showed no significant difference in fold change of cycle frequency between OrgR, OrgR>shi^{ts}, HugS3>shi^{ts} (performed Mann-Whitney Rank Sum Test (n.s. – not significant)).
(TIF)

Figure S6 (A,B) Statistical analysis of food intake and wandering-like behavior assay of OrgR×shi^{ts} compared to HugS3>shi^{ts} under starved (A) and fed (B) conditions. The graph (left) shows the

intake of yeast (area of the alimentary tract stained by colored yeast divided by body surface area) after 20 min at 32°C. Graph (right) illustrates the statistical data of the wandering-like behavior of OrgR×shi^{ts} compared to HugS3>shi^{ts} measured as larvae outside the yeast/min [%] over a time period of 20 min. In both nutritional conditions HugS3>shi^{ts} showed no significant difference in food intake and wandering-like behavior relative to OrgR×shi^{ts} at 32°C.
(TIF)

Figure S7 Hugin antibody staining of the genotypes: OrgR>dTrpA1, HugS3>dTrpA1, HugS3>dTrpA1, HugRNAi1A and HugS3>dTrpA1, TRiP.JF03122. Images show the subesophageal ganglion of the larval CNS as indicated in the schematic drawing (left side, scale bar: 20 μm).
(TIF)

Figure S8 (A) Hugin antibody staining of hug0.8>rpr/hid showing four remaining cells in the SOG that project to the VNC (A, scale bar: 50 μm; A′, scale bar: 10 μm).
(TIF)

Figure S9 Graphs show the relative change in cycle frequency of M6- and CDM-motor pattern of HugS3>dTrpA1 and TRH>dTrpA1 compared to the control lines (Mann-Whitney Rank Sum Test: *p≤0.05, **p≤0.01, ***p≤0.001).
(TIF)

Acknowledgments

We thank Rachel Wilson and Ansgar Büschges for help with electrophysiology, Loren Looger, Stefan Pulver, Hermann Aberle, Leslie Griffith, Paul Garrity, Ping Shen, Julie Simpson, Jay Hirsh, Olga Alekseyenko, David Krantz, Gero Miesenböck, Hiromu Tanimoto, and the Bloomington and Kyoto Stock Centers for reagents, Anja Nagel and Anette Preiss for RNAi vector, Silvana Opp and Thor Kastilan for RNAi construct, SFB 645 and 704, NRW LIMES graduate school, DFG Cluster of Excellence ImmunoSensation, DFG grant PA 787 for financial support, and Ingo Zinke, Ravi Allada, Valerie Kilman, and Frank Hirth for valuable discussions, and Claire McKellar for suggesting the SOG–brainstem analogy. We also thank Gaia Tavosanis and R.W. for critical comments on earlier versions of this manuscript.

Author Contributions

The author(s) have made the following declarations about their contributions: Conceived and designed the experiments: AS SH PS AM MP MJP. Performed the experiments: AS SH PS AM MP MZ. Analyzed the data: AS SH PS AM MP MZ MJP. Contributed reagents/materials/analysis tools: RS ASC. Wrote the paper: AS MJP.

References

1. Grillner S, Hellgren J, Ménard A, Saitoh K, Wikström MA (2005) Mechanisms for selection of basic motor programs—roles for the striatum and pallidum. Trends Neurosci 28: 364–370.
2. Delcomyn F (1980) Neural basis of rhythmic behavior in animals. Science 210: 492–498.
3. Harris-Warwick RM, Marder E, Selverston AI, Moulins M (1992) Dynamic biological networks: the stomatogastric nervous system. Cambridge (Massachusetts): The MIT Press.
4. Grillner S (2003) The motor infrastructure: from ion channels to neuronal networks. Nat Rev Neurosci 4: 573–586.
5. Grillner S (2006) Biological pattern generation: the cellular and computational logic of networks in motion. Neuron 52: 751–766.
6. Grillner S, Jessell TM (2009) Measured motion: searching for simplicity in spinal locomotor networks. Curr Opin Neurobiol 19: 572–586.
7. Goulding M (2009) Circuits controlling vertebrate locomotion: moving in a new direction. Nat Rev Neurosci 10: 507–518.
8. Kiehn O, Kullander K (2004) Central pattern generators deciphered by molecular genetics. Neuron 41: 317–321.
9. Büschges A, Scholz H, El Manira A (2011) New moves in motor control. Curr Biol 21: R513–R524.
10. Kiehn O (2011) Development and functional organization of spinal locomotor circuits. Curr Opin Neurobiol 21: 100–109.
11. Whelan PJ (2010) Shining light into the black box of spinal locomotor networks. Philos Trans R Soc Lond B Biol Sci 365: 2383–2395.
12. Takakusaki K, Saitoh K, Harada H, Kashiwayanagi M (2004) Role of basal ganglia-brainstem pathways in the control of motor behaviors. Neurosci Res 50: 137–151.
13. Cazalets J, Borde M, Clarac F (1995) Localization and organization of the central pattern generator for hindlimb locomotion in newborn rat. J Neurosci 15 (7): 4943–4951.
14. Jiang Z, Carlin KP, Brownstone RM (1999) An in vitro functionally mature mouse spinal cord preparation for the study of spinal motor networks. Brain Res 816: 493–499.
15. Gosgnach S, Lanuza GM, Butt SJB, Saueressig H, Zhang Y, et al. (2006) V1 spinal neurons regulate the speed of vertebrate locomotor outputs. Nature 440: 215–219.
16. Miles GB, Hartley R, Todd AJ, Brownstone RM (2007) Spinal cholinergic interneurons regulate the excitability of motoneurons during locomotion. Proc Natl Acad Sci U S A 104: 2448–2453.
17. Smetana R, Juvin L, Dubuc R, Alford S (2010) A parallel cholinergic brainstem pathway for enhancing locomotor drive. Nat Neurosci 13: 731–738.
18. Wyart C, Del Bene F, Warp E, Scott EK, Trauner D, et al. (2009) Optogenetic dissection of a behavioural module in the vertebrate spinal cord. Nature 461: 407–410.
19. Zagoraiou L, Akay T, Martin JF, Brownstone RM, Jessell TM, et al. (2009) A cluster of cholinergic premotor interneurons modulates mouse locomotor activity. Neuron 64: 645–662.
20. Miri A, Azim E, Jessell TM (2013) Edging toward entelechy in motor control. Neuron 80: 827–834.
21. Marder E, Bucher D (2007) Understanding circuit dynamics using the stomatogastric nervous system of lobsters and crabs. Annu Rev Physiol 69: 291–316.
22. Hirayama K, Catanho M, Brown JW, Gillette R (2012) A core circuit module for cost/benefit decision. Front Neurosci 6: 123.
23. Melcher C, Bader R, Pankratz MJ (2007) Amino acids, taste circuits, and feeding behavior in *Drosophila*: towards understanding the psychology of feeding in flies and man. J Endocrinol 192: 467–472.
24. Palmer CR, Kristan WB (2011) Contextual modulation of behavioral choice. Curr Opin Neurobiol 21: 520–526.
25. Taghert PH, Nitabach MN (2012) Peptide neuromodulation in invertebrate model systems. Neuron 76: 82–97.
26. Belgardt BF, Brüning JC (2010) CNS leptin and insulin action in the control of energy homeostasis. Ann N Y Acad Sci 1212: 97–113.
27. Oury F, Karsenty G (2011) Towards a serotonin-dependent leptin roadmap in the brain. Trends Endocrinol Metab 22: 382–387.
28. Sokolowski MB (1980) Foraging strategies of *Drosophila melanogaster*: a chromosomal analysis. Behav Genet 10: 291–302.
29. Avery L, Horvitz HR (1989) Pharyngeal pumping continues after laser killing of the pharyngeal nervous system of *C. elegans*. Neuron 3: 473–485.
30. Avery L (1993) The Genetics of Feeding in *Caenorhabditis elegans*. Genetics 133: 897–917.
31. Sokolowski MB (2001) *Drosophila*: genetics meets behaviour. Nat Rev Genet 2: 879–890.
32. Avery L, You YJ (2012) *C. elegans* feeding. WormBook: 1–23.
33. Buch S, Pankratz MJ (2009) Making metabolic decisions in *Drosophila*. Fly (Austin) 3: 74–77.
34. Flood TF, Iguchi S, Gorczyca M, White B, Ito K, et al. (2013) A single pair of interneurons commands the *Drosophila* feeding motor program. Nature 499: 83–87.
35. Gordon MD, Scott K (2009) Motor control in a *Drosophila* taste circuit. Neuron 61: 373–384.
36. Hergarden AC, Tayler TD, Anderson DJ (2012) Allatostatin-A neurons inhibit feeding behavior in adult *Drosophila*. Proc Natl Acad Sci U S A 109: 3967–3972.
37. Manzo A, Silies M, Gohl DM, Scott K (2012) Motor neurons controlling fluid ingestion in *Drosophila*. Proc Natl Acad Sci U S A 109: 6307–6312.
38. Marella S, Mann K, Scott K (2012) Dopaminergic modulation of sucrose acceptance behavior in *Drosophila*. Neuron 73: 941–950.
39. Mann K, Gordon MD, Scott K (2013) A pair of interneurons influences the choice between feeding and locomotion in *Drosophila*. Neuron 79: 754–765.
40. Olsen SR, Wilson RI (2008) Cracking neural ciruits in a tiny brain: new approach for understanding the neural circuits of *Drosophila*. Trends Neurosci 31: 512–520.
41. Simpson JH (2009) Mapping and manipulating neural circuits in the fly brain. Adv Genet 65: 79–143.
42. Scott K (2005) Taste recognition: food for thought. Neuron 48: 455–464.
43. Vosshall LB, Stocker RF (2007) Molecular architecture of smell and taste in *Drosophila*. Annu Rev Neurosci 30: 505–533.
44. Su C-Y, Menuz K, Carlson JR (2009) Olfactory perception: receptors, cells, and circuits. Cell 139: 45–59.

45. Schoofs A, Niederegger S, van Ooyen A, Heinzel H-G, Spiess R (2010) The brain can eat: establishing the existence of a central pattern generator for feeding in third instar larvae of *Drosophila virilis* and *Drosophila melanogaster*. J Insect Physiol 56: 695–705.
46. Sarov-Blat L, So WV, Liu L, Rosbash M (2000) The *Drosophila* takeout gene is a novel molecular link between circadian rhythms and feeding behavior. Cell 101: 647–656.
47. Zinke I, Kirchner C, Chao LC, Tetzlaff MT, Pankratz MJ (1999) Suppression of food intake and growth by amino acids in *Drosophila*: the role of pumpless, a fat body expressed gene with homology to vertebrate glycine cleavage system. Development 126: 5275–5284.
48. Gutierrez E, Wiggins D, Fielding B, Gould AP (2007) Specialized hepatocyte-like cells regulate *Drosophila* lipid metabolism. Nature 445: 275–280.
49. Melcher C, Pankratz MJ (2005) Candidate gustatory interneurons modulating feeding behavior in the *Drosophila* brain. PLoS Biol 3: e305.
50. Nakazato M, Hanada R, Murakami N, Date Y, Mondal MS, et al. (2000) Central effects of neuromedin U in the regulation of energy homeostasis. Biochem Biophys Res Commun 277: 191–194.
51. Nixon JF, Kotz CM, Novak CM, Billington CJ, Teske JA (2012) Neuropeptides Controlling Energy Balance: Orexins and Neuromedins. Handb Exp Pharmacol 209: 77–109.
52. Hamada FN, Rosenzweig M, Kang K, Pulver SR, Ghezzi A, et al. (2008) An internal thermal sensor controlling temperature preference in *Drosophila*. Nature 454: 217–222.
53. Mahr A, Aberle H (2006) The expression pattern of the *Drosophila* vesicular glutamate transporter: a marker protein for motoneurons and glutamatergic centers in the brain. Gene Expr Patterns 6: 299–309.
54. Luan H, Lemon WC, Peabody NC, Pohl JB, Zelensky PK, et al. (2006) Functional dissection of a neuronal network required for cuticle tanning and wing expansion in *Drosophila*. J Neurosci 26: 573–584.
55. Nitabach MN, Wu Y, Sheeba V, Lemon WC, Strumbos J, et al. (2006) Electrical hyperexcitation of lateral ventral pacemaker neurons desynchronizes downstream circadian oscillators in the fly circadian circuit and induces multiple behavioral periods. J Neurosci 26: 479–489.
56. Kitamoto T (2001) Conditional modification of behavior in *Drosophila* by targeted expression of a temperature-sensitive shibire allele in defined neurons. J Neurobiol 47: 81–92.
57. Buch S, Melcher C, Bauer M, Katzenberger J, Pankratz MJ (2008) Opposing effects of dietary protein and sugar regulate a transcriptional target of *Drosophila* insulin-like peptide signaling. Cell Metab 7: 321–332.
58. Ataman B, Ashley J, Gorczyca M, Ramachandran P, Fouquet W, et al. (2008) Rapid activity-dependent modifications in synaptic structure and function require bidirectional Wnt signaling. Neuron 57: 705–718.
59. Jan LY, Jan YN (1976) Properties of the larval neuromuscular junction in *Drosophila melanogaster*. J Physiol 262: 189–214.
60. Cattaert D, Birman S (2001) Blockade of the central generator of locomotor rhythm by noncompetitive NMDA receptor antagonists in *Drosophila* larvae. J Neurobiol 48: 58–73.
61. Barclay JW, Atwood HL, Robertson RM (2002) Impairment of central pattern generation in *Drosophila* cysteine string protein mutants. J Comp Physiol A 188: 71–78.
62. Calabrese RL (1998) Cellular, synaptic, network, and modulatory mechanisms involved in rhythm generation. Curr Opin Neurobiol 8: 710–717.
63. Marder E, Calabrese RL (1996) Principles of rhythmic motor pattern generation. Physiol Rev 76: 687–717.
64. Bader R, Colomb J, Pankratz B, Schröck A, Stocker RF, et al. (2007) Genetic dissection of neural circuit anatomy underlying feeding behavior in *Drosophila*: distinct classes of hugin- expressing neurons. J Comp Neurol 856: 848–856.
65. Kristan WB (2008) Neuronal decision-making circuits. Curr Biol 18: R928–32.
66. Spieß R, Schoofs A, Heinzel HG (2008) Anatomy of the stomatogastric nervous system associated with the foregut in *Drosophila melanogaster* and *Calliphora vicina* third instar larvae. J Morphol 269: 272–282.
67. Lahiri S, Shen K, Klein M, Tang A, Kane E, et al. (2011) Two alternating motor programs drive navigation in *Drosophila* larva. PLoS One 6: e23180.
68. Berni J, Pulver SR, Griffith LC, Bate M (2012) Autonomous circuitry for substrate exploration in freely moving *Drosophila* larvae. Curr Biol 22: 1861–1870.
69. Vallés AM, White K (1986) Development of serotonin-containing neurons in *Drosophila* mutants unable to synthesize serotonin. J Neurosci 6: 1482–1491.
70. Miller AJ (1993) The search for the central swallowing pathway: the quest for clarity. Dysphagia 8: 185–194.
71. Jean A (2001) Brain stem control of swallowing: neuronal network and cellular mechanisms. Physiol Rev 81: 929–969.
72. Bieger D, Neuhuber W (2006) Neural circuits and mediators regulating swallowing in the brainstem. Gastrointest Motil Online. doi: 10.1038/gimo74
73. Williams KW, Elmquist JK (2012) From neuroanatomy to behavior: central integration of peripheral signals regulating feeding behavior. Nat Neurosci 15: 1350–1355.
74. Shepherd GM (1994) Neurobiology. 3rd Edition. New York (NY): Oxford University Press Inc.
75. Strausfeld NJ, Hirth F (2013) Deep homology of arthropod central complex and vertebrate basal ganglia. Science 340: 157–161.
76. Stephenson-Jones M, Samuelsson E, Ericsson J, Robertson B, Grillner S (2011) Evolutionary conservation of the basal ganglia as a common vertebrate mechanism for action selection. Curr Biol 21: 1081–1091.
77. Wessnitzer J, Webb B (2006) Multimodal sensory integration in insects—towards insect brain control architectures. Bioinspir Biomim 1: 63–75.
78. Bader R, Sarraf-Zadeh L, Peters M, Moderau N, Stocker H, et al. (2013) The IGFBP7 homolog Imp-L2 promotes insulin signaling in distinct neurons of the *Drosophila* brain. J Cell Sci 126: 2571–2576.
79. Rosenkilde C, Cazzamali G, Williamson M, Hauser F, Søndergaard L, et al. (2003) Molecular cloning, functional expression, and gene silencing of two *Drosophila* receptors for the *Drosophila* neuropeptide pyrokinin-2. Biochem Biophys Res Commun 309: 485–494.
80. Park Y, Kim Y-J, Adams ME (2002) Identification of G protein-coupled receptors for *Drosophila* PRXamide peptides, CCAP, corazonin, and AKH supports a theory of ligand-receptor coevolution. Proc Natl Acad Sci U S A 99: 11423–11428.
81. Cardona A, Saalfeld S, Preibisch S, Schmid B, Cheng A, et al. (2010) An integrated micro- and macroarchitectural analysis of the *Drosophila* brain by computer-assisted serial section electron microscopy. PLoS Biol 8: e1000502.
82. White JG, Southgate E, Thomson JN, Brenner S (1986) The structure of the nervous system of the nematode *Caenorhabditis elegans*. Philos Trans R Soc London B 314: 1–340.
83. Ng M, Roorda RD, Lima SQ, Zemelman B V, Morcillo P, et al. (2002) Transmission of olfactory information between three populations of neurons in the antennal lobe of the fly. Neuron 36: 463–474.
84. Alekseyenko O V, Lee C, Kravitz EA (2010) Targeted manipulation of serotonergic neurotransmission affects the escalation of aggression in adult male *Drosophila melanogaster*. PLoS One 5: e10806.
85. Rulifson EJ, Kim SK, Nusse R (2002) Ablation of insulin-producing neurons in flies: growth and diabetic phenotypes. Science 296: 1118–1120.
86. Vosshall LB, Wong a M, Axel R (2000) An olfactory sensory map in the fly brain. Cell 102: 147–159.
87. Pfeiffer BD, Jenett A, Hammonds AS, Ngo T-TB, Misra S, et al. (2008) Tools for neuroanatomy and neurogenetics in *Drosophila*. Proc Natl Acad Sci U S A 105: 9715–9720.
88. Rohrbough J, Broadie K (2002) Electrophysiological analysis of synaptic transmission in central neurons of *Drosophila* larvae. J Neurophysiol 88: 847–860.
89. Nagel AC, Maier D, Preiss A (2002) Green fluorescent protein as a convenient and versatile marker for studies on functional genomics in *Drosophila*. Dev Genes Evol 212: 93–98.
90. Rubin GM, Spradling AC (1982) Genetic transformation of *Drosophila* with transposable element vectors. Science 218: 348–353.
